# Supplementary material for: A Case for Automated Segmentation of MRI Data in Neurodegenerative Diseases: Type II GM1 Gangliosidosis
Source: NeuroSci. 2025 Apr 3;6(2):31. doi: 10.3390/neurosci6020031 (PMC12015847; doi:10.3390/neurosci6020031)
Supplement: Supplementary file 1 [file neurosci-06-00031-s001.zip › neurosci-3515731-supplementary.pdf]

A Case for Automated Segmentation of MRI Data in Neurodegenerative Diseases: Type II GM1 Gangliosidosis
Supplementary Material

Table of Contents

Methods……………………………………………………………………………..……….*2*

S1: Natural History Study Participant Characteristics…..…………..……...…….……..*2*

S2: Late-Infantile GM1 Patients Age Matched Methodology…………….…….……..*3*

S3: Juvenile GM1 Patients Age Matched Methodology…………………….………...*4*

S4: MRI Scan Exclusion……………………………………………..…………..*6*

Results……………………………………………………………………………..…......*9*

S5. Cross-sectional analysis of Juvenile Patients ……………………...…………*9*

S6. Cross-sectional analysis of Late-Infantile Patients…………………………………..…*14*

S7. Correlation Strength Tables between Manual and Automated Segmentation……*19*

S8. Correlations between Manual and Freesurfer Segmentation……………….………*22*

S9. Correlations between Manual and volBrain Segmentation……………...……….…..*25*

S10. Correlations between Manual and FSL Segmentation………………...……...…..*28*

S11. Correlations between Manual and SPM Segmentation…………….……………….*31*

S12. Correlations between Manual and Headreco Segmentation……………….……….*33*

S13. Correlation Data Slope Estimates and Intercepts………………………….……….*36*

S14. Supplementary References……...………………………...………………......*38*

Supplementary Methods

S1: Natural History Study Participant Characteristics[1]

Table S1.1. Natural History Study T1-Weighted MRI Cohort (n = 24)

Participant	GM1 Sub-type	Age of Symptom Onset (years)	Age of Diagnosis (years)	Baseline Scan Age (years)	Scan #2 Age (years)	Scan #3 Age (years)	Scan #4 Age (years)	Average interval between imaging (years)	Number of T1 Scans
NHS 13	LI	1.5	4.0	4.8	N/A	N/A	N/A	N/A	1
NHS 24	LI	0.9	1.3	6.9	N/A	N/A	N/A	N/A	1
NHS 35	LI	2.5	2.5	5.0	N/A	N/A	N/A	N/A	1
NHS 63	LI	1.5	2.0	3.5	N/A	N/A	N/A	N/A	1
NHS 72	LI	1.5	6.0	8.8	9.8	N/A	N/A	0.95	2
NHS 73	LI	1.5	3.0	5.9	7.0	N/A	N/A	1.1	2
NHS 84	LI	1.1	2.3	3.2	4.2	N/A	N/A	1.0	2
NHS 85	LI	1.0	3.0	6.1	N/A	N/A	N/A	N/A	1
NHS 03	Juv	5.0	11.0	23.3	24.5	25.3	N/A	1.0	3
NHS 09	Juv	3.0	12.0	13.4	15.6	19.8	21.8	2.8	4
NHS 10	Juv	2.5	11.0	13.3	15.5	19.7	22.8	3.2	4
NHS 11	Juv	2.5	9.0	12.3	15.8	17.8	19.9	2.5	4
NHS 20	Juv	5.0	10.0	12.0	13.1	15.5	N/A	1.8	3
NHS 25	Juv	4.0	14.0	12.2	14.8	19.3	N/A	3.6	3
NHS 26	Juv	3.0	11.0	12.0	13.5	16.6	N/A	2.3	3
NHS 27	Juv	4.0	6.0	7.5	12.0	N/A	N/A	4.5	2
NHS 28	Juv	5.0	19.0	19.2	20.2	22.5	N/A	1.7	3
NHS 54	Juv	2.5	5.5	7.4	8.3	9.3	N/A	1.0	3
NHS 58	Juv	4.0	11.0	14.6	16.1	17.2	N/A	1.3	3
NHS 69	Juv	1.5	3.0	7.0	8.2	9.9	N/A	1.5	3
NHS 71	Juv	2.5	10.0	13.2	N/A	N/A	N/A	N/A	1
NHS 93	Juv	3.0	8.0	9.7	10.7	N/A	N/A	1.0	2
NHS 94	Juv	3.0	8.0	9.1	10.1	N/A	N/A	1.0	2
NHS 95	Juv	asymptomatic	1.0	2.4	3.7	N/A	N/A	1.3	2

S2: Age-Matched Methodology for Late-Infantile Patients

To evaluate the five automated segmentation pipelines' ability to demonstrate volumetric differences between the late-infantile patients and neurotypical controls, we performed a cross-sectional analysis including age and sex-matched data (Table B1 and B2). Seven late-infantile patients' (4 females, 3 males) baseline MRI scans and 7 neurotypical control (4 females, 3 males) scans from the Calgary Preschool[2] and Adolescent[3] databases were included in this analysis.

Table S2.1. Late-infantile Age Matched Cohort

Participant	Sex	Age
NHS 13	F	4.8
NHS 35	F	5.0
NHS 63	M	3.5
NHS 72	M	8.8
NHS 73	M	5.9
NHS 84	F	3.2
NHS 85	F	6.1
		5.3 ± 1.9

Table S2.2. Late-infantile Age Matched Cohort of Neurotypical Controls

Participant	Dataset	Sex	Age
Calgary 10014	Preschool	M	3.6
Calgary 10027	Preschool	F	4.0
Calgary 10044	Preschool	M	3.4
Calgary 10064	Preschool	F	2.7
Calgary 15003	Adolescent	F	8.3
Calgary 15008	Adolescent	F	11.1
Calgary 15010	Adolescent	M	9.1
			6.0 ± 3.4

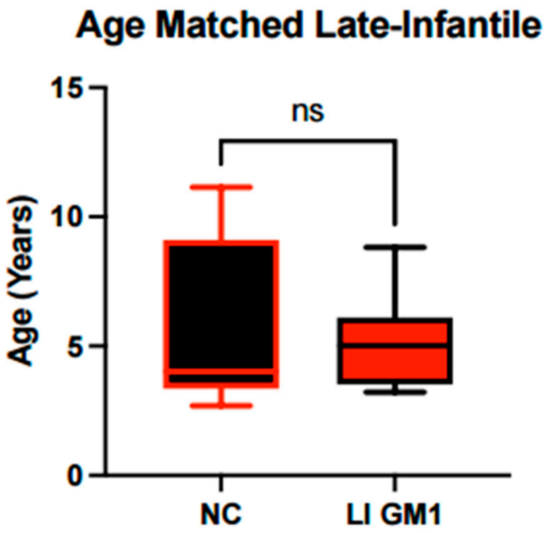

Figure S2.1. Age and sex-matched late-infantile GM1 gangliosidosis cohort evaluated for differences by a two tailed t-test (t=0.4771, df =12, p = 0.6418).

S3: Age-Matched Methodology for Juvenile Patients

To evaluate the 5 automated segmentation pipelines' ability to demonstrate volumetric differences between the juvenile patients and neurotypical controls, we performed a cross-sectional analysis including age and sex-matched data (Table C1 and C2). Fourteen juvenile patients' (8 females, 6 males) baseline MRI scans and 7 neurotypical control (4 females, 3 males) scans from the Calgary Preschool[2] and Adolescent[3] databases were included in this analysis.

Table S3.1. Juvenile Age Matched Cohort

Participant	Sex	Age
NHS 09	M	11-15
NHS 10	F	11-15
NHS 11	M	11-15
NHS 20	F	11-15
NHS 25	F	11-15
NHS 26	M	11-15
NHS 27	M	6-10
NHS 28	F	16-20
NHS 54	F	6-10
NHS 58	F	11-15
NHS 69	F	6-10
NHS 93	F	6-10
NHS 94	M	6-10
NHS 95	M	0-5
		10.9 ± 4.1

Table S3.2. Juvenile Age Matched Cohort of Neurotypical Controls

Participant	Dataset	Sex	Age
Calgary 10027	Preschool	F	0-5
Calgary 14006	Preschool	M	6-10
Calgary 14008	Preschool	M	11-15
Calgary 14011	Preschool	M	11-15
Calgary 14013	Adolescent	F	11-15
Calgary 15003	Adolescent	F	6-10
Calgary 15008	Adolescent	F	11-15
			10.6 ± 2.9

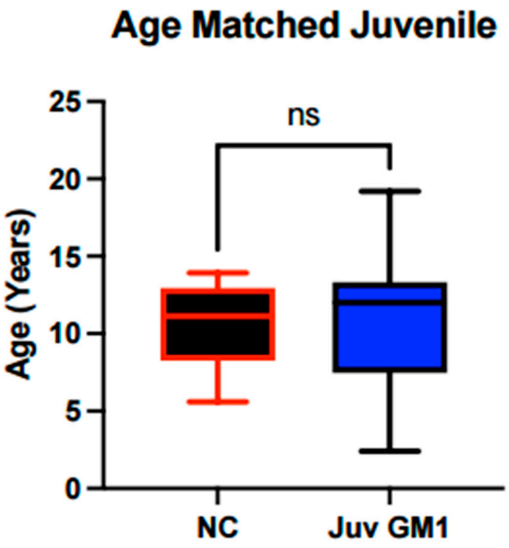

Figure S3.1. Age and sex-matched juvenile GM1 gangliosidosis cohort evaluated for differences by a 2 tailed *t*-test (t=0.1757, df =19, p = 0.8624).

S4: MRI Exclusion

Manual Segmentation Process
All 19 MRI scans from neurotypical controls, all 11 scans from 8 late-infantile GM1 gangliosidosis patients, and all 45 MRI scans from 16 juvenile GM1 gangliosidosis patients were analyzed using the manual segmentation method.

Freesurfer
All 19 MRI scans from 11 neurotypical controls were analyzed using the Freesurfer *recon-all* pipeline. 10 MRI scans from 7 late-infantile patients were analyzed using the Freesurfer *recon-all* pipeline, with one late-infantile patient MRI scan failing to process due to significant atrophy and demyelination (Figure D1). Thirty-nine MRI scans from 16 juvenile patients were analyzed using the Freesurfer *recon-all* pipeline with one 3 MRI scans failing to process due to the presence of bone marrow in the cerebrospinal fluid (Figure D2) and 3 MRI scans failing to process due to significant atrophy (Figure D3).

volBrain
All 19 MRI scans from 11 neurotypical controls were analyzed using volBrain's *vol2Brain* pipeline. Ten MRI scans from 7 late-infantile patients were analyzed using the volBrain's *vol2Brain* pipeline, with one late-infantile patient MRI scan failing to process due to significant atrophy and demyelination (Figure D1). All 45 MRI scans from 16 juvenile GM1 gangliosidosis patients were analyzed using the volBrain's *vol2Brain* segmentation method.

FSL
FSL FAST
All 19 MRI scans from 11 neurotypical controls were analyzed using FSL's *FAST* pipeline. Ten MRI scans from 7 late-infantile GM1 gangliosidosis patients were analyzed using FSL's *FAST* pipeline with one late-infantile patient MRI scan failing to process due to significant atrophy and demyelination (Figure D1). All 45 MRI scans from 16 juvenile GM1 gangliosidosis patients were analyzed using the FSL's *fast* segmentation method.

FSL FIRST
All 19 MRI scans from 11 neurotypical controls were analyzed using the FSL's *FIRST* pipeline. 10 MRI scans from 7 late-infantile patients were analyzed using FSL's *FIRST* with one late-infantile patient MRI scan failing to process due to significant atrophy (Figure D1). Forty-three MRI scans from 16 juvenile GM1 gangliosidosis patients were analyzed using the FSL *first* segmentation methods. Two MRI scans failed to process due to the presence of bone marrow in the cerebrospinal fluid (Figure D2) and significant atrophy (Figure D3, Scan3).

SPM12
All 19 MRI scans from 11 neurotypical controls were analyzed using the SPM12's *Segment* pipeline. All 11 MRI scans from late-infantile GM1 gangliosidosis patients were analyzed using SPM12's *Segment* pipeline. All 45 MRI scans from 16 juvenile GM1 gangliosidosis patients were analyzed using the SPM12's *Segment* segmentation method.

SimNIBS' Headreco

All 19 MRI scans from 11 neurotypical controls were analyzed using the SimNIBS' *Headreco* pipeline. Ten MRI scans from 7 late-infantile patients were analyzed using the SimNIBS' *Headreco* pipeline, with one late-infantile patient MRI scan failing to process due to significant atrophy and demyelination (Figure S4.1.). Forty-three scans from 16 juvenile GM1 patients were analyzed using SimNIBS. Two scans from a juvenile participant of the GM1 gangliosidosis cohort were excluded from this analysis due to the presence of bone marrow in the cerebrospinal fluid (Figure S4.2.).

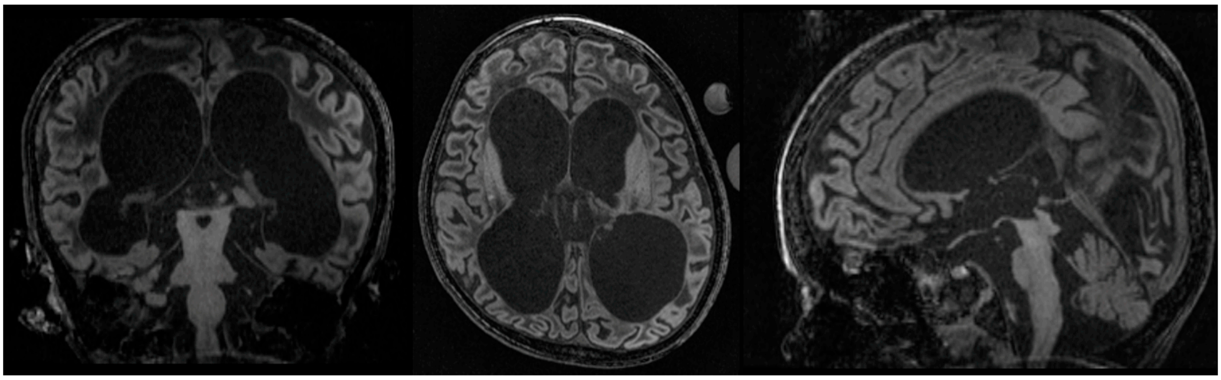

Figure S4.1. Coronal, Axial, and Sagittal T1-weighted MRI of a late-infantile GM1 patient with significant cerebral atrophy, ventricle enlargement, and hypomyelination.

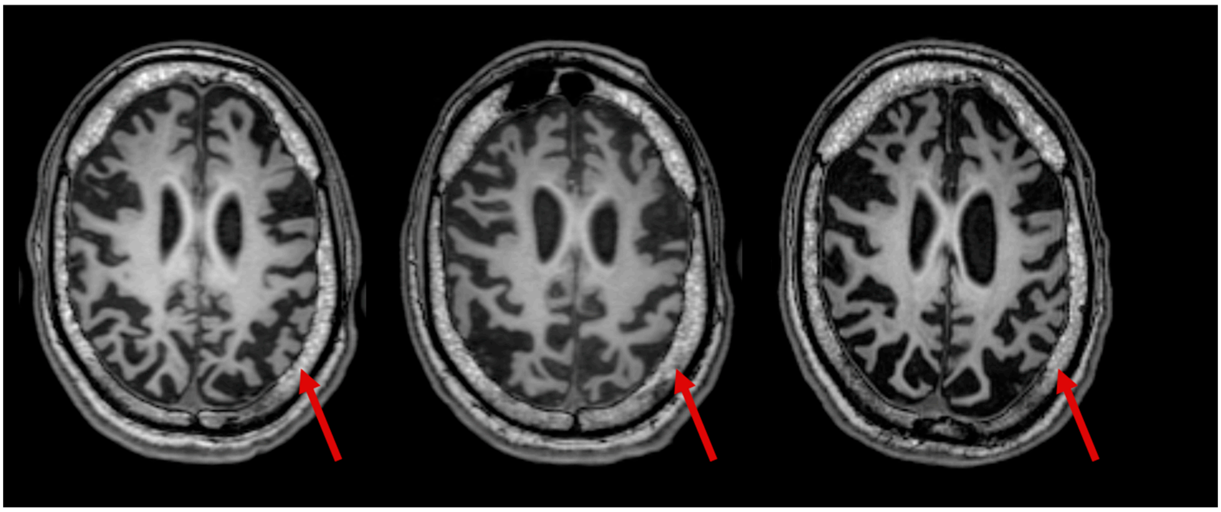

Figure S4.2. Serial T1 MRI of an excluded juvenile GM1 patient at age 11-15 (Scan 1), 16-20 (Scan 2), and 16-20 (Scan 3) years old with bone marrow (red arrow) in the cerebrospinal fluid.

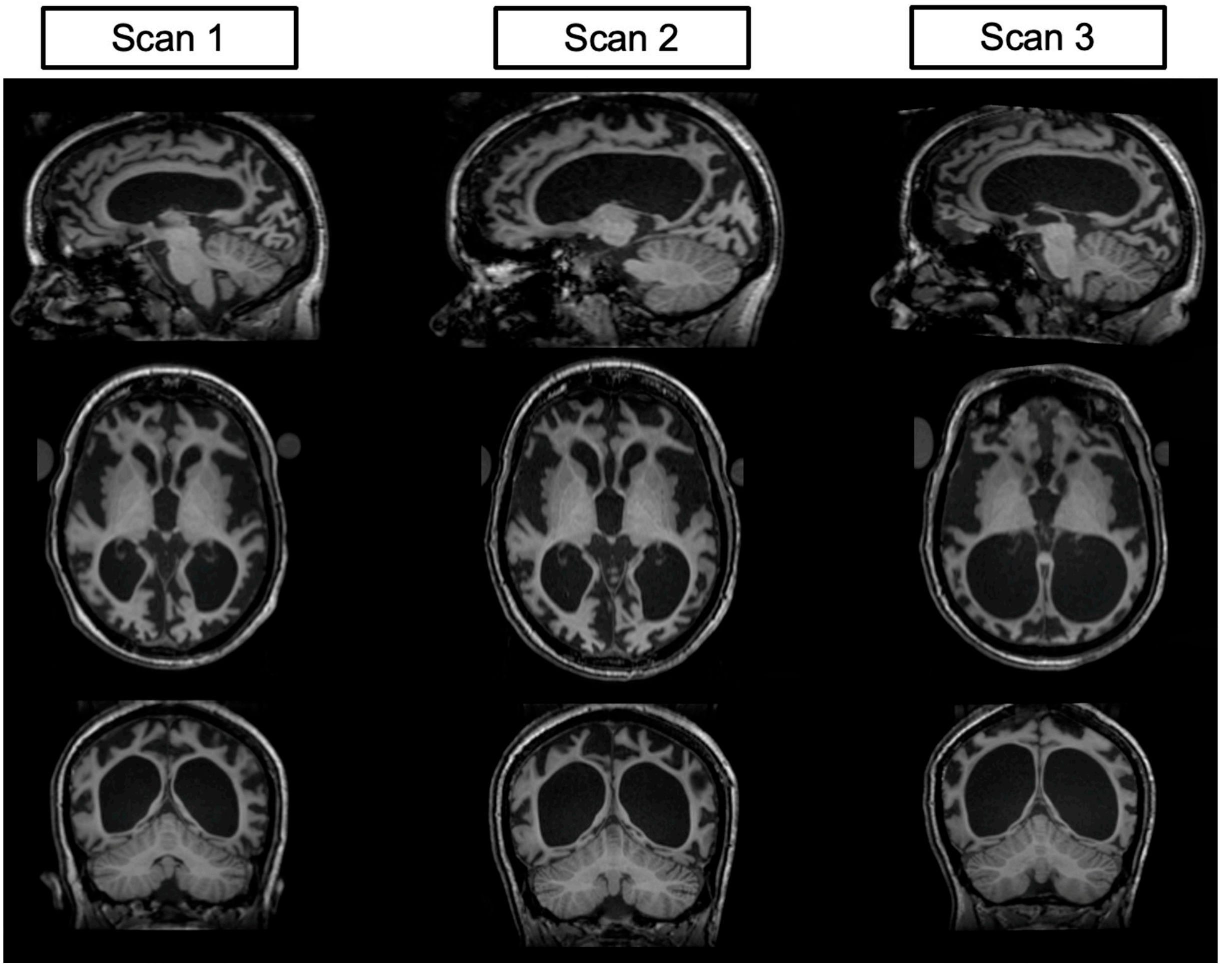

Figure S4.3. Serial T1 MRI imaging of a juvenile GM1 patient at age 16-20 (Scan 1), 16-20 (Scan 2), and 21-25 (Scan 3) years old with significant atrophy and ventricle enlargement across three evaluations.

S5. Cross-sectional Analysis of Juvenile Patients

Ventricles

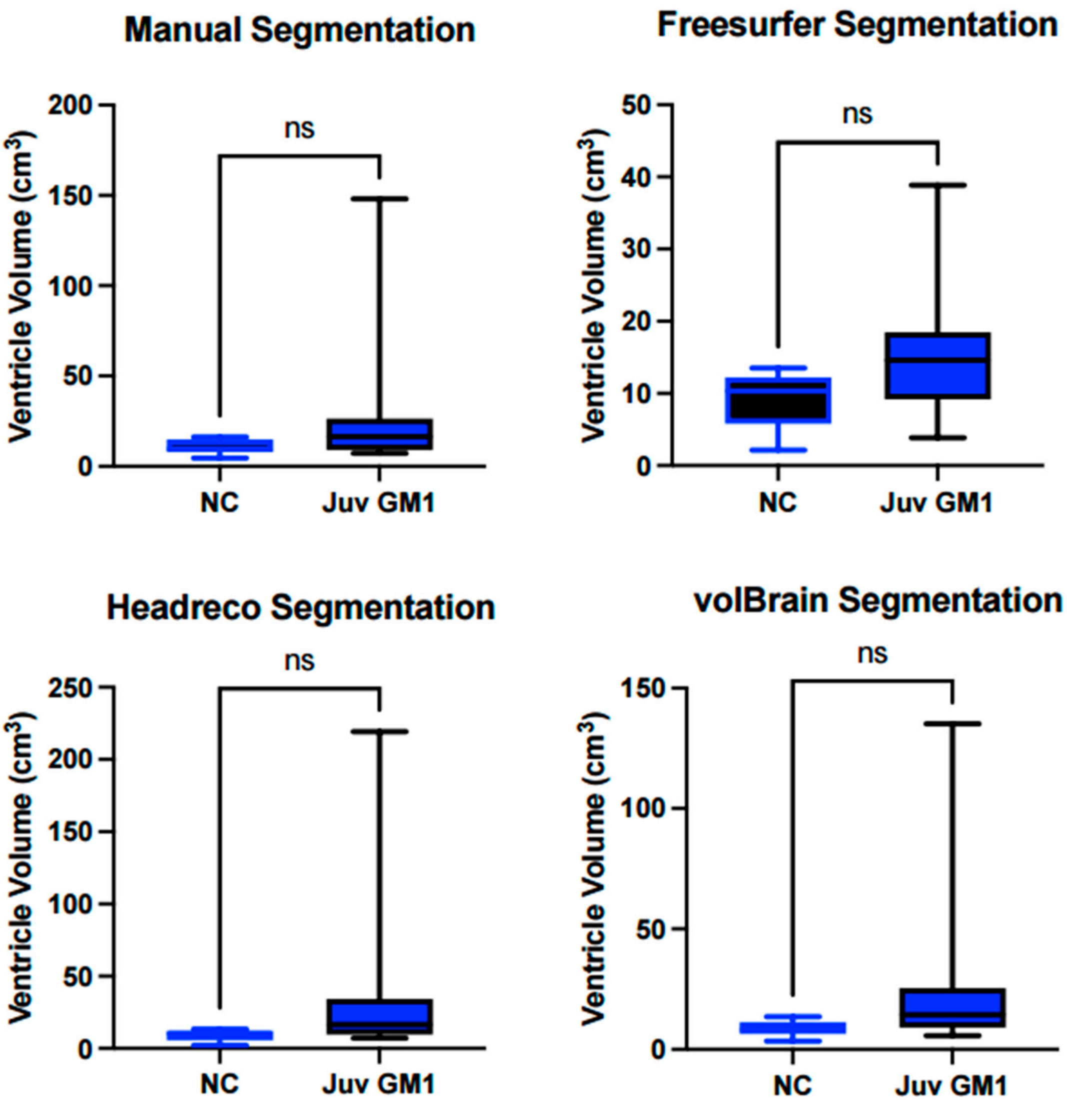

Figure S5.1. Cross-sectional evaluation of the 3 automated segmentation algorithms to demonstrate cohort differences in ventricle volume. Juvenile (Juv) GM1 patients (n = 14) are shown in blue. Neurotypical controls (NC, n = 7) are shown in black. *P*-values were calculated from the *t*-statistic. * $P < 0.05$, ** $P < 0.01$, *** $P < 0.001$, **** $P < 0.0001$.

Cerebellum

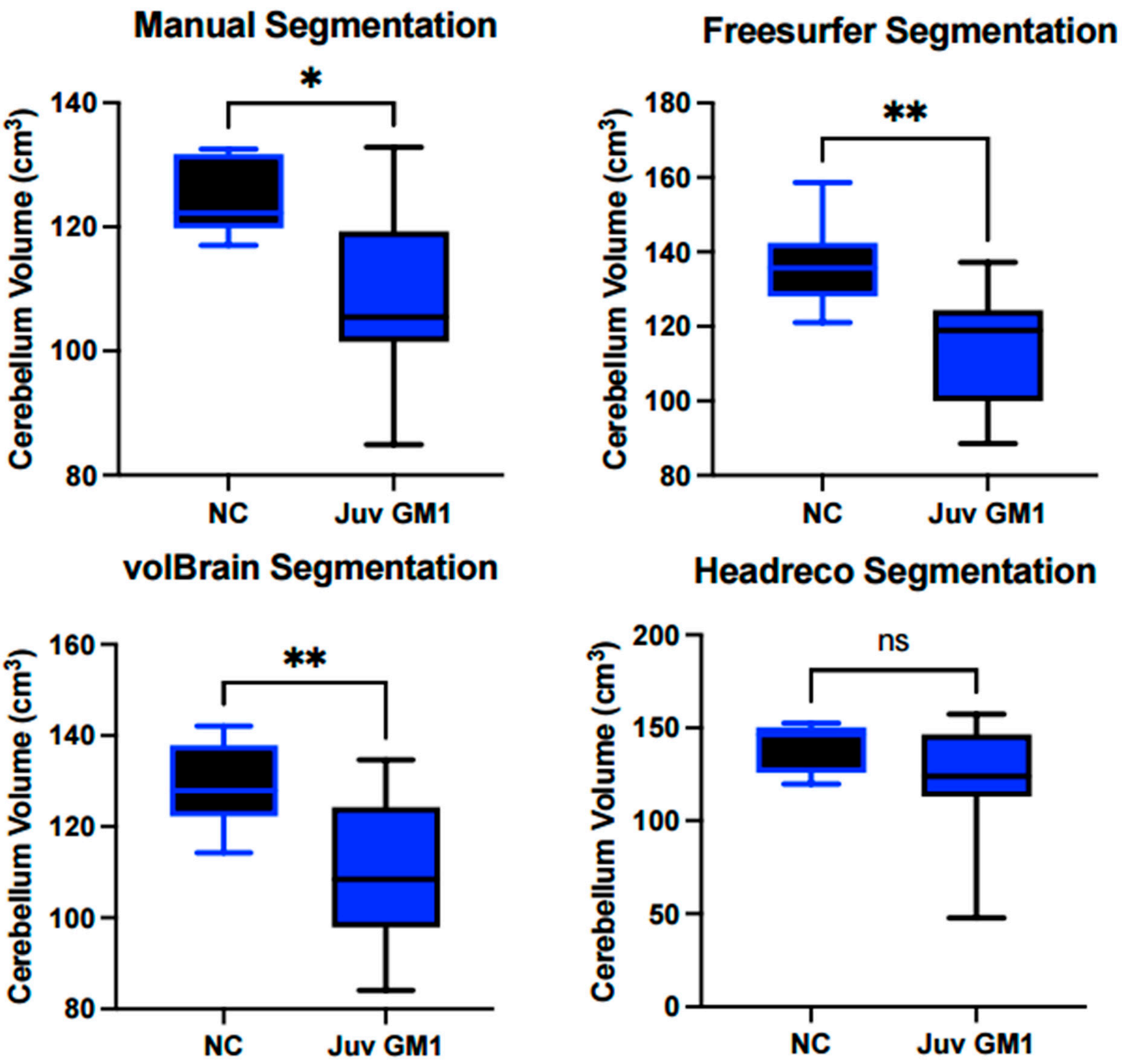

Figure S5.2. Cross-sectional evaluation of the 3 automated segmentation algorithms to demonstrate cohort differences in cerebellum volume. Juvenile (Juv) GM1 patients (n = 14) are shown in blue. Neurotypical controls (NC, n = 7) are shown in black. *P*-values were calculated from the *t*-statistic. * $P < 0.05$, ** $P < 0.01$, *** $P < 0.001$, **** $P < 0.0001$.

Caudate Nucleus

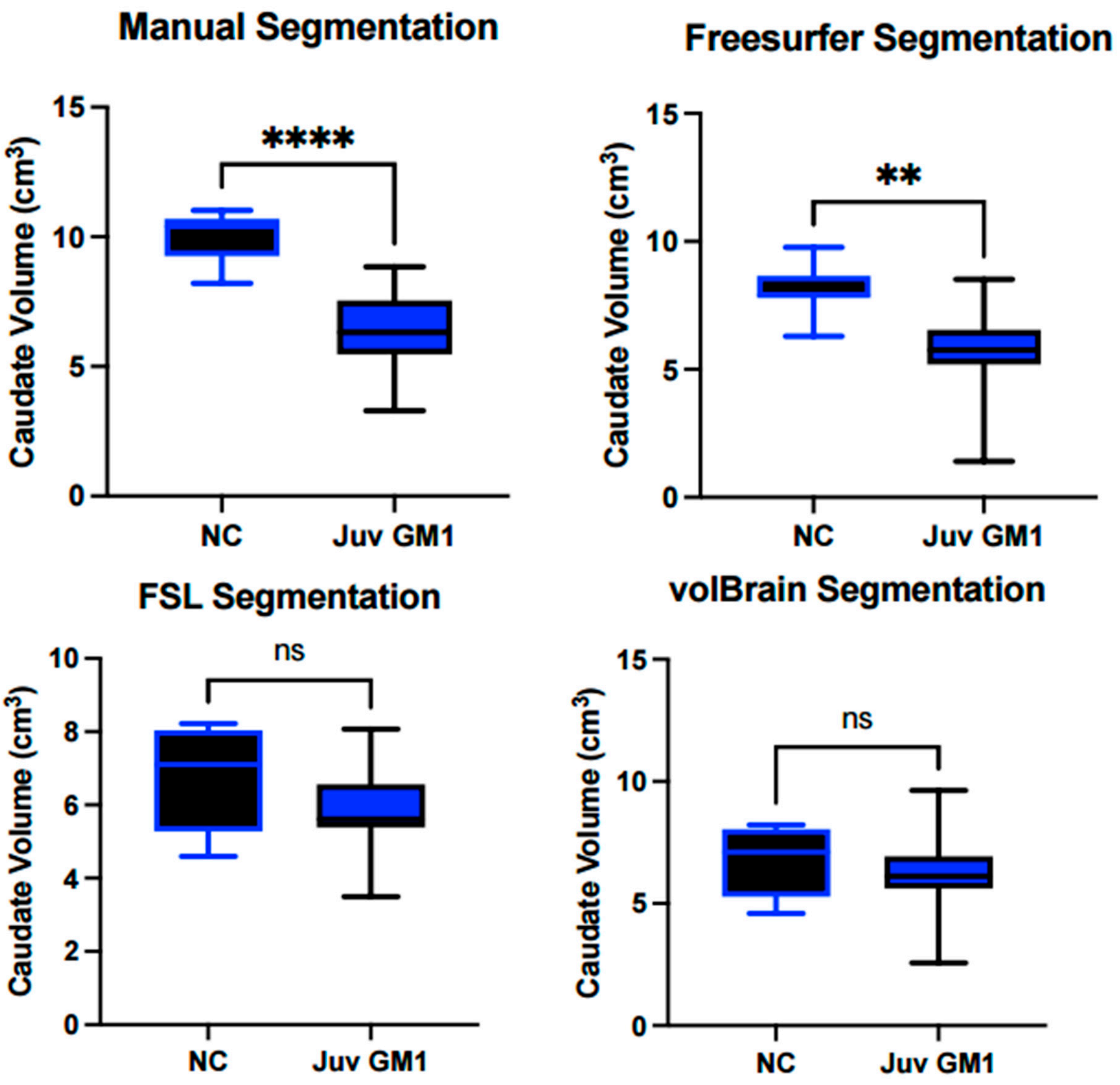

Figure S5.3. Cross-sectional evaluation of the 3 automated segmentation algorithms to demonstrate cohort differences in caudate volume. Juvenile (Juv) GM1 patients (n = 14) are shown in blue. Neurotypical controls (NC, n = 7) are shown in black. *P*-values were calculated from the *t*-statistic. * $P < 0.05$, ** $P < 0.01$, *** $P < 0.001$, **** $P < 0.0001$.

Lentiform Nucleus

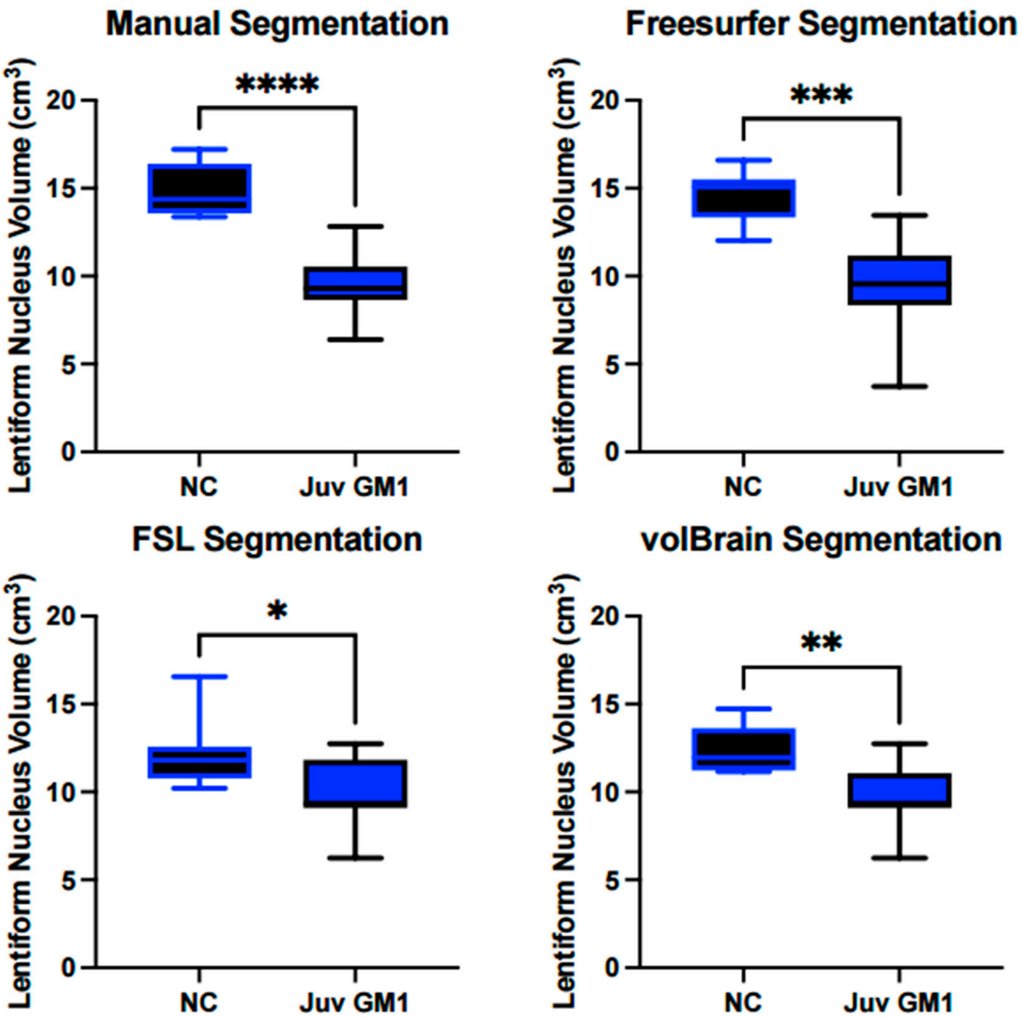

Figure S5.4. Cross-sectional evaluation of the 3 automated segmentation algorithms to demonstrate cohort differences in lentiform nucleus volume. Juvenile (Juv) GM1 patients (n = 14) are shown in blue. Neurotypical controls (NC, n = 7) are shown in black. *P*-values were calculated from the *t*-statistic. * $P < 0.05$, ** $P < 0.01$, *** $P < 0.001$, **** $P < 0.0001$.

Corpus Callosum

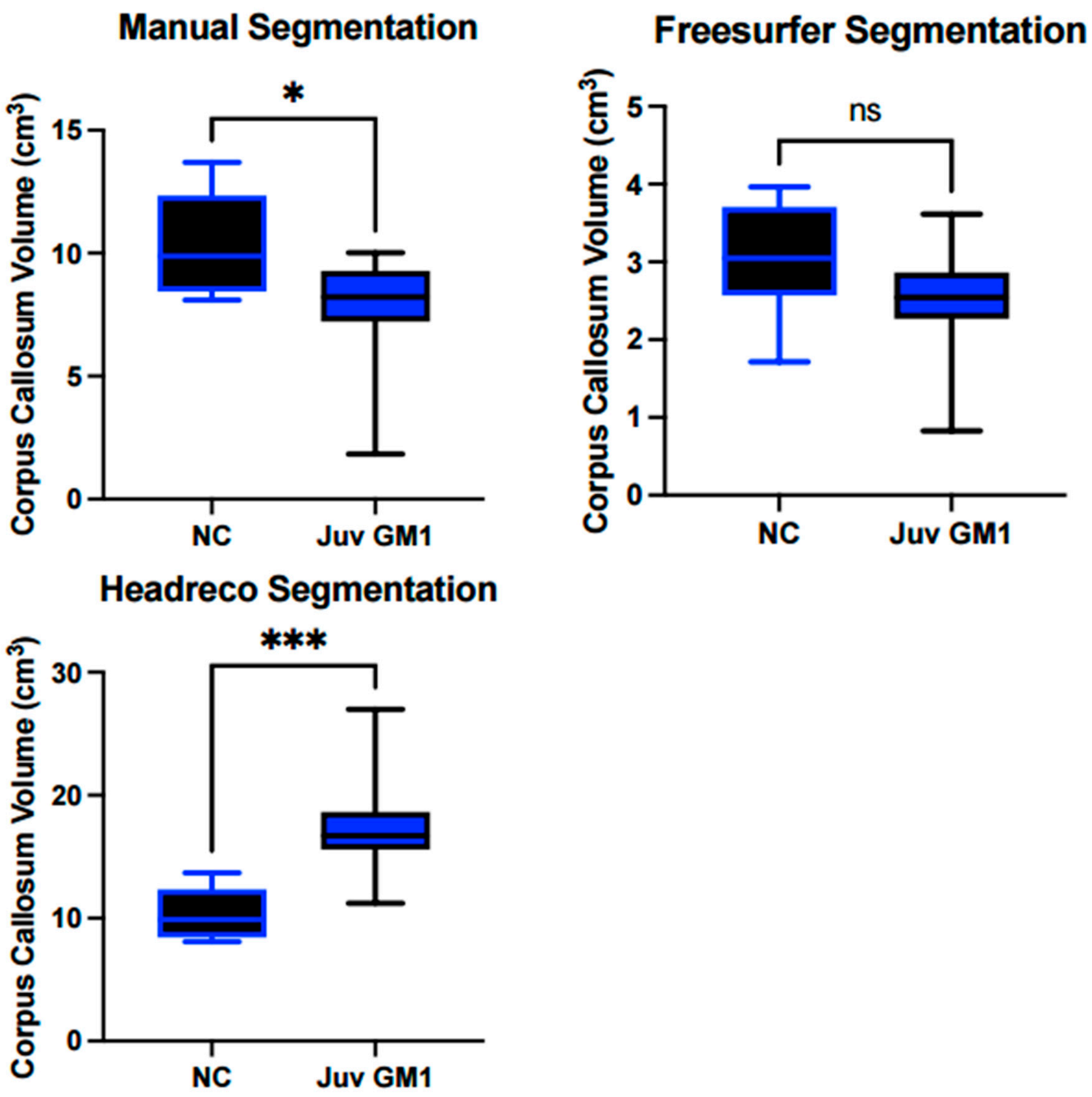

Figure S5.5. Cross-sectional evaluation of the 2 automated segmentation algorithms to demonstrate cohort differences in corpus callosum volume. Juvenile (Juv) GM1 patients (n = 14) are shown in blue. Neurotypical controls (NC, n = 7) are shown in black. *P*-values were calculated from the *t*-statistic. * $P < 0.05$, ** $P < 0.01$, *** $P < 0.001$, **** $P < 0.0001$.

S6. Cross-sectional Analysis of Late-Infantile Patients

Cerebellum

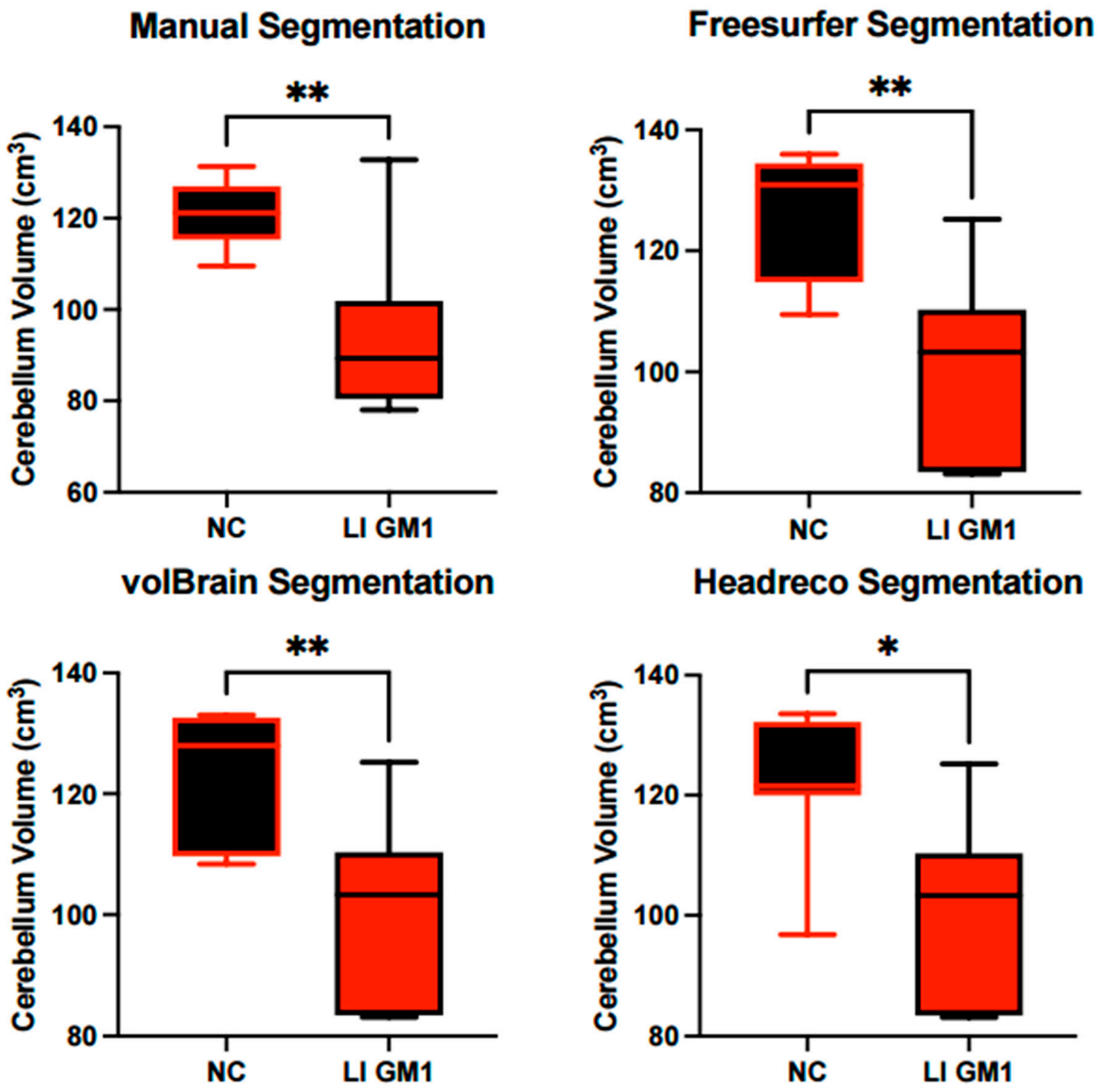

Figure S6.1. Cross-sectional evaluation of the 3 automated segmentation algorithms to demonstrate cohort differences in cerebellar volume. Late-infantile (LI) GM1 patients (n = 7) are shown in Red. Neurotypical controls (NC, n = 7) are shown in black. *P*-values were calculated from the *t*-statistic. * $P < 0.05$, ** $P < 0.01$, *** $P < 0.001$, **** $P < 0.0001$.

Thalamus

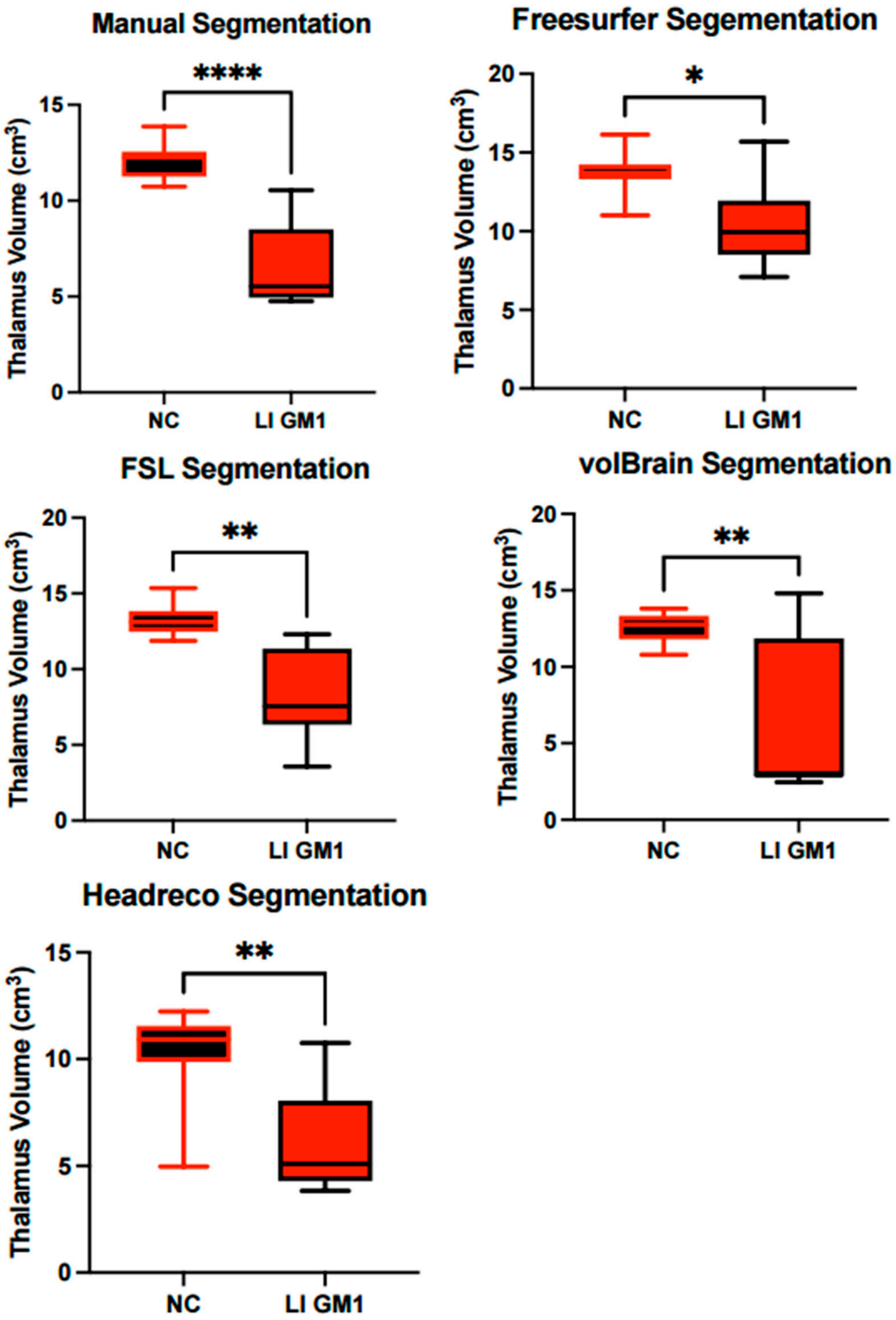

Figure S6.2. Cross-sectional evaluation of the 4 automated segmentation algorithms to demonstrate cohort differences in thalamic volume. Late-infantile (LI) GM1 patients (n = 7) are shown in Red. Neurotypical controls (NC, n = 7) are shown in black. *P*-values were calculated from the *t*-statistic. * $P < 0.05$, ** $P < 0.01$, *** $P < 0.001$, **** $P < 0.0001$.

Caudate Nucleus

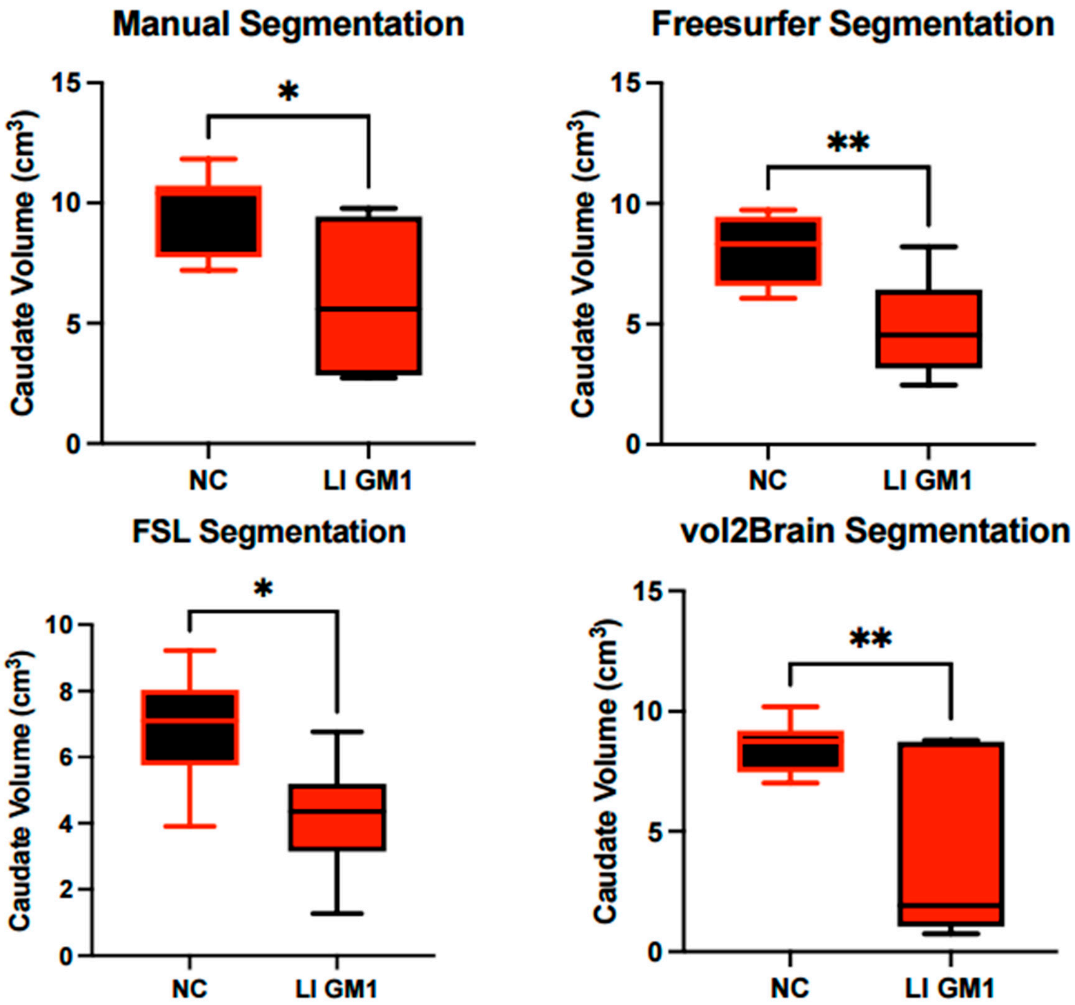

Figure S6.3. Cross-sectional evaluation of the 3 automated segmentation algorithms to demonstrate cohort differences in caudate volume. Late-infantile (LI) GM1 patients (n = 7) are shown in Red. Neurotypical controls (NC, n = 7) are shown in black. *P*-values were calculated from the *t*-statistic. * $P < 0.05$, ** $P < 0.01$, *** $P < 0.001$, **** $P < 0.0001$.

Lentiform Nucleus

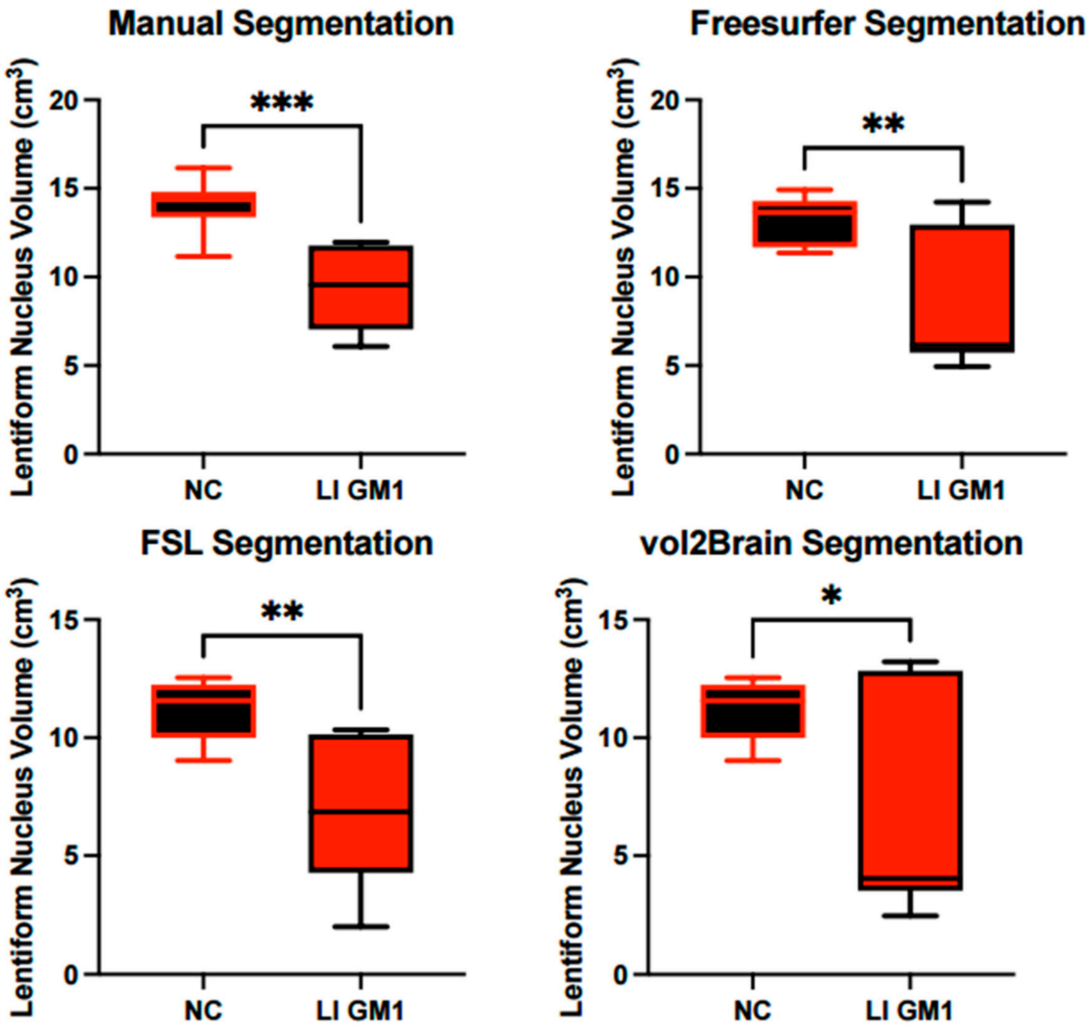

Figure S6.4. Cross-sectional evaluation of the 3 automated segmentation algorithms to demonstrate cohort differences in lentiform nucleus volume. Late-infantile (LI) GM1 patients (n = 7) are shown in Red. Neurotypical controls (NC, n = 7) are shown in black. *P*-values were calculated from the *t*-statistic. * $P < 0.05$, ** $P < 0.01$, *** $P < 0.001$, **** $P < 0.0001$.

Corpus Callosum

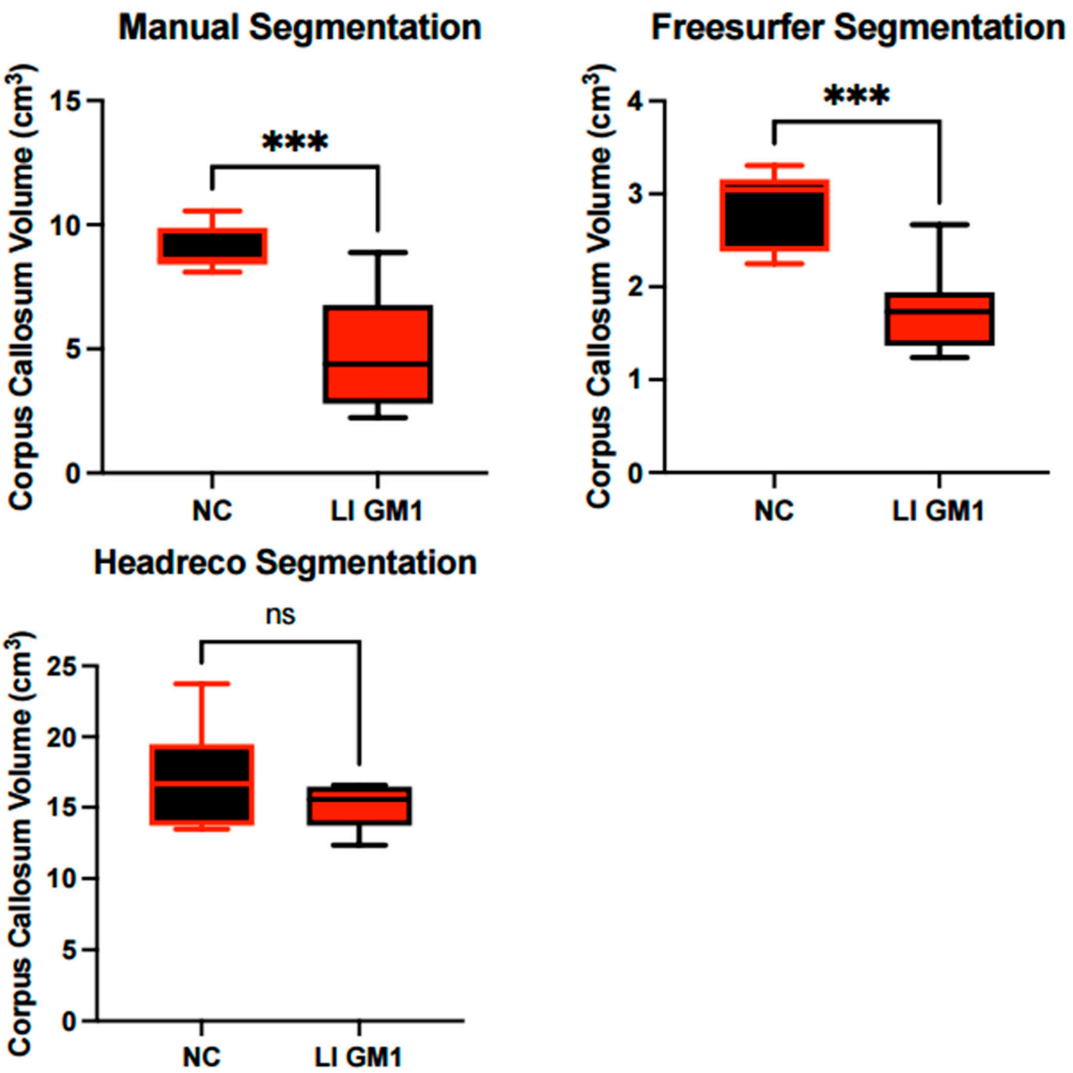

Figure S6.5. Cross-sectional evaluation of the 2 automated segmentation algorithms to demonstrate cohort differences in corpus callosum volume. Late-infantile (LI) GM1 patients (n = 7) are shown in Red. Neurotypical controls (NC, n = 7) are shown in black. *P*-values were calculated from the *t*-statistic. * $P < 0.05$, ** $P < 0.01$, *** $P < 0.001$, **** $P < 0.0001$.

S7: Correlation Strength Tables between Manual and Automated Segmentation

Table S7.1. Comparison of correlation coefficients (R^2, top row) between the 5 fully automated segmentation pipelines and the manual pipeline in the 7 different brain regions for the neurotypical controls. Normality was determined using the Shapiro-Wilk test. *P*-values (bottom row) were calculated from the Pearson product moment correlation coefficient when the data was normally distributed and from Spearman's rank correlation coefficient when the data was not normally distributed and designated with a $^+$. N/A are designations where the region was not calculated using the specified segmentation algorithm.

Structure	Freesurfer	FSL	volBrain	SPM	SimNIBS Headreco (SPM+CAT)
**Whole Brain**	0.8256<br>$p < 0.0001$	0.9058<br>$p < 0.0001$	0.9852<br>$p < 0.0001$	0.8629<br>$p < 0.0001^+$	0.8593<br>$p < 0.0001$
**Ventricles**	0.6180<br>$p < 0.0001$	N/A	0.9452<br>$p < 0.0001$	N/A	0.7951<br>$p < 0.0001$
**Cerebellum**	0.6910<br>$p < 0.0001$	N/A	0.8269<br>$p < 0.0001$	N/A	0.5384<br>$p = 0.0003$
**Thalamus**	0.5359<br>$p = 0.0004$	0.5074<br>$p = 0.0006$	0.6276<br>$p < 0.0001$	N/A	0.5322<br>$p < 0.0001^+$
**Caudate**	0.8174<br>$p < 0.0001$	0.5416<br>$p = 0.0003$	0.7282<br>$p < 0.0001$	N/A	N/A
**Lentiform Nucleus**	0.7251<br>$p < 0.0001$	0.5271<br>$p = 0.0004^+$	0.5562<br>$p = 0.0002$	N/A	N/A
**Corpus Callosum**	0.06547<br>$p = 0.2904$	N/A	N/A	N/A	0.4829<br>$p = 0.0010$

Table S7.2. Comparison of correlation coefficients (R^2, top row) between the 5 fully automated segmentation pipelines and the manual pipeline in the 7 different brain regions for the juvenile GM1 patients. Normality was determined using the Shapiro-Wilk test. *P*-values (bottom row) were calculated from the Pearson product moment correlation coefficient when the data was normally distributed and from Spearman's rank correlation coefficient when the data was not normally distributed and designated with a $^+$. N/A are designations where the region was not calculated using the specified segmentation algorithm.

**Structure**	**Freesurfer**	**FSL**	**volBrain**	**SPM**	**SimNIBS Headreco (SPM+CAT)**
**Whole Brain**	0.9196<br>$p < 0.0001^+$	0.6038<br>$p < 0.0001$	0.9583<br>$p < 0.0001^+$	0.9642<br>$p < 0.0001^+$	0.8956<br>$p < 0.0001^+$
**Ventricles**	0.9765<br>$p < 0.0001^+$	N/A	0.9782<br>$p < 0.0001^+$	N/A	0.7422<br>$p < 0.0001^+$
**Cerebellum**	0.9133<br>$p < 0.0001$	N/A	0.9258<br>$p < 0.0001$	N/A	0.6009<br>$p < 0.0001^+$
**Thalamus**	0.4359<br>$p < 0.0001$	0.5033<br>$p < 0.0001^+$	0.8053<br>$p < 0.0001^+$	N/A	0.7073<br>$p < 0.0001^+$
**Caudate**	0.8181<br>$p < 0.0001^+$	0.5068<br>$p < 0.0001^+$	0.8885<br>$p < 0.0001^+$	N/A	N/A
**Lentiform Nucleus**	0.5660<br>$p < 0.0001$	0.4266<br>$p < 0.0001$	0.6605<br>$p < 0.0001$	N/A	N/A
**Corpus Callosum**	0.4050<br>$p = 0.0122^+$	N/A	N/A	N/A	0.3131<br>$p < 0.0001$

Table S7.3. Comparison of correlation coefficients (R^2, top row) between the 5 fully automated segmentation pipelines and the manual pipeline in the 7 different brain regions for the late-infantile GM1 patients. Normality was determined using the Shapiro-Wilk test. *P*-values (bottom row) were calculated from the Pearson product moment correlation coefficient when the data was normally distributed and from Spearman's rank correlation coefficient when the data was not normally distributed and designated with a $^+$. N/A are designations where the region was not calculated using the specified segmentation algorithm.

Structure	Freesurfer	FSL	volBrain	SPM	SimNIBS Headreco (SPM+CAT)
**Whole Brain**	0.3460<br>$p = 0.0736$	0.6585<br>$p = 0.0044$	0.9169<br>$p = 0.0072^+$	0.5458<br>$p = 0.0094$	0.8246<br>$p = 0.0003$
**Ventricles**	0.7467<br>$p = 0.0013$	N/A	0.9974<br>$p < 0.0001$	N/A	0.9950<br>$p < 0.0001$
**Cerebellum**	0.6728<br>$p = 0.0037$	N/A	0.8374<br>$p < 0.0001$	N/A	0.8779<br>$p < 0.0001$
**Thalamus**	0.5347<br>$p = 0.0163$	0.6585<br>$p = 0.0044$	0.8632<br>$p < 0.0001^+$	N/A	0.7809<br>$p = 0.0003$
**Caudate**	0.8536<br>$p = 0.0001$	0.2584<br>$p = 0.1335$	0.7394<br>$p = 0.0027^+$	N/A	N/A
**Lentiform Nucleus**	0.6619<br>$p = 0.0347^+$	0.7713<br>$p = 0.0008$	0.6518<br>$p = 0.0368^+$	N/A	N/A
**Corpus Callosum**	0.06030<br>$p = 0.4941$	N/A	N/A	N/A	< 0.01<br>$p = 0.9895^+$

S8: Correlations between Manual and Freesurfer Segmentation

Neurotypical Controls

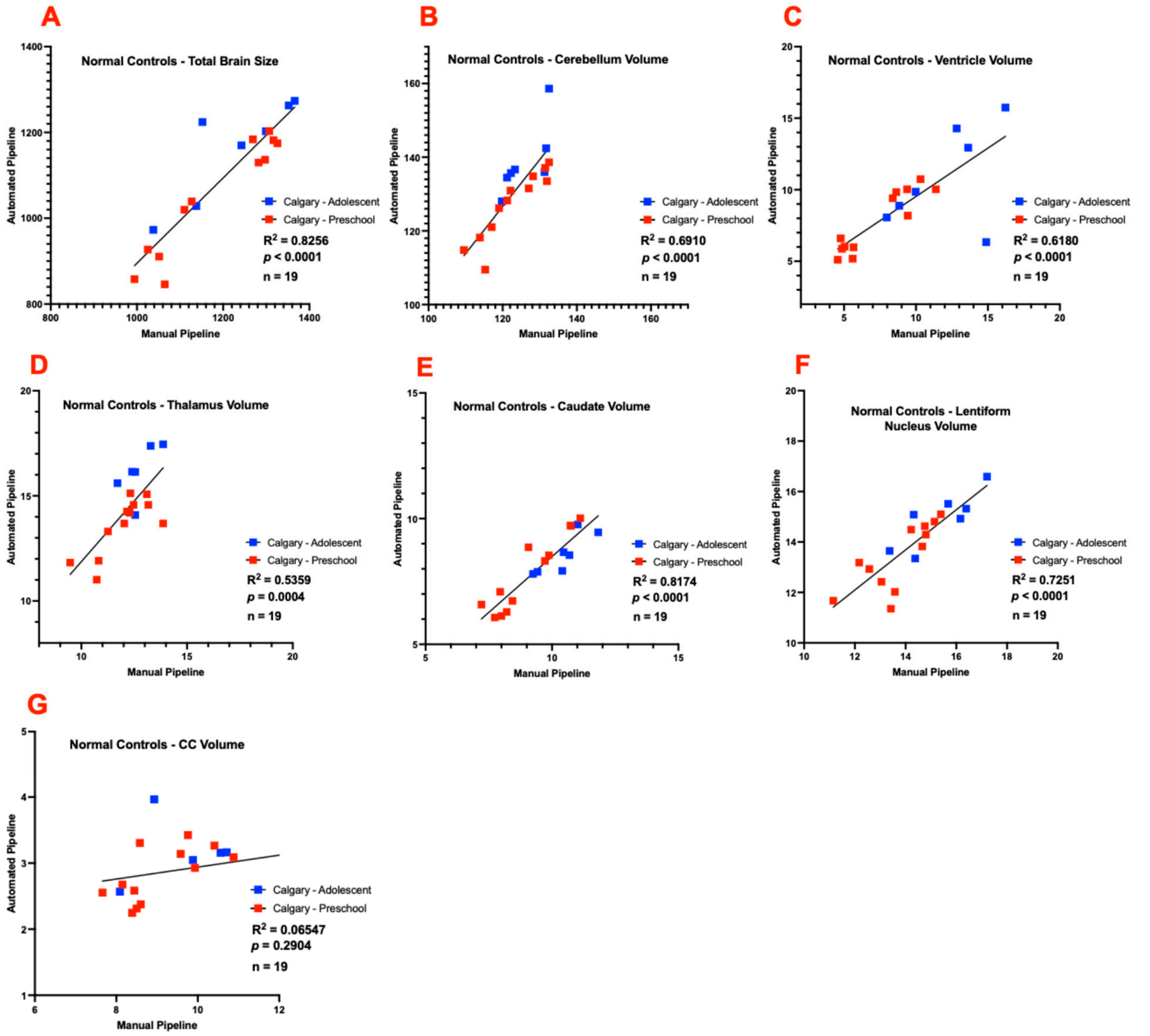

Figure S8.1. Correlations between Freesurfer's automated and the manual approach in neurotypical controls for the A.) Total brain volume B.) Cerebellum volume C.) Ventricle volume D.) Thalamic volume E.) Caduate volume F.) Lentiform nucleus volume and G.) Corpus callosum volume. Participants from the Calgary preschool MRI data set are shown in red and participants from the adolescent Calgary dataset are shown in blue. All volumes are shown in cm^3.

Juvenile GM1 Patients

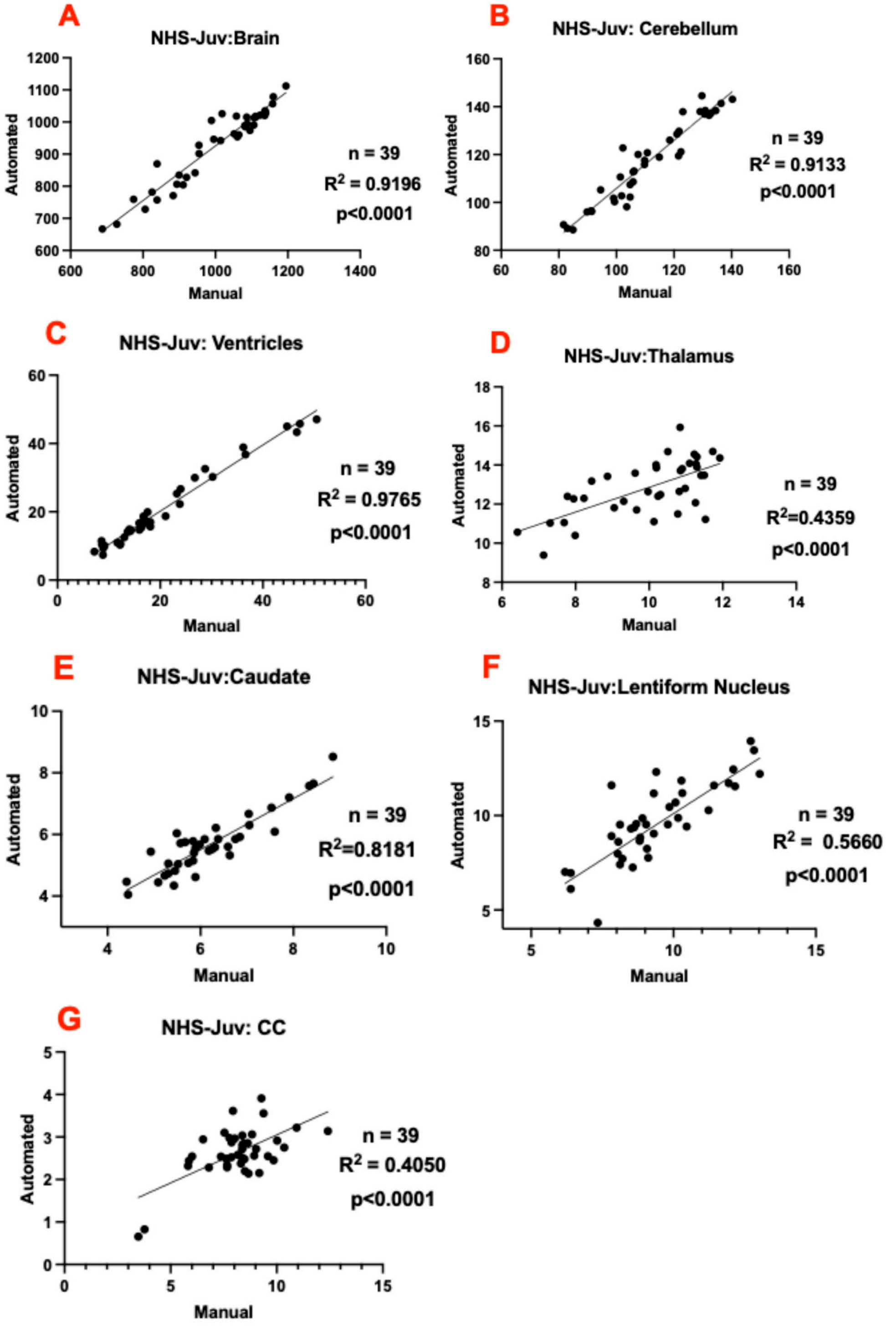

Figure S8.2. Correlations between Freesurfer's automated and the manual approach in natural history study (NHS) juvenile GM1 gangliosidosis patients for the A.) Total brain volume B.) Cerebellum volume C.) Ventricle volume D.) Thalamic volume E.) Caduate volume F.) Lentiform nucleus volume and G.) Corpus callosum volume. All volumes are shown in cm^3.

Late-infantile GM1 Patients

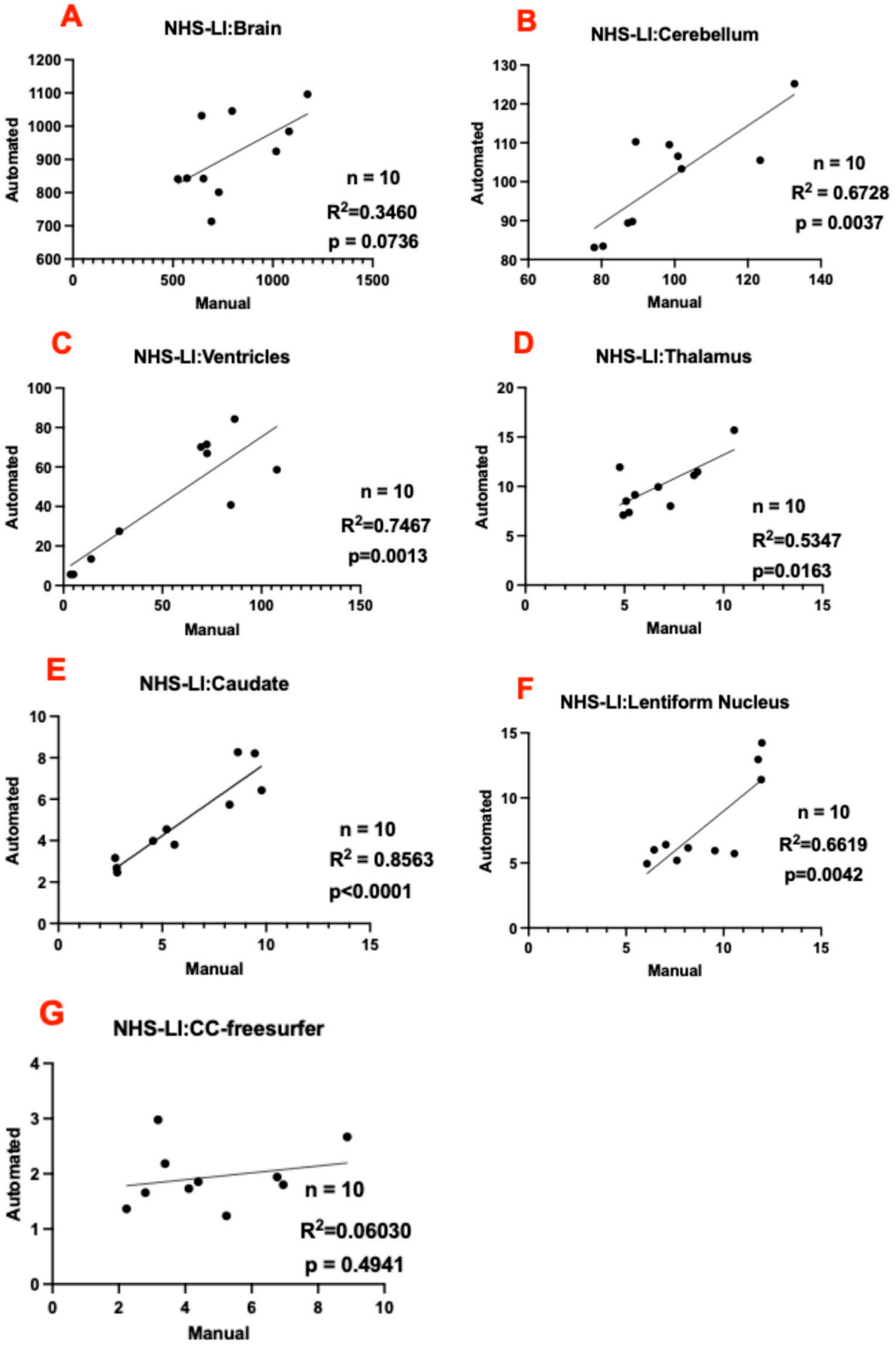

Figure S8.3. Correlations between Freesurfer's automated and the manual approach in natural history study (NHS) late-infantile GM1 gangliosidosis patients for the A.) Total brain volume B.) Cerebellum volume C.) Ventricle volume D.) Thalamic volume E.) Caduate volume F.) Lentiform nucleus volume and G.) Corpus callosum volume. All volumes are shown in cm^3.

S9: Correlations between Manual and volBrain Segmentation

Neurotypical Controls

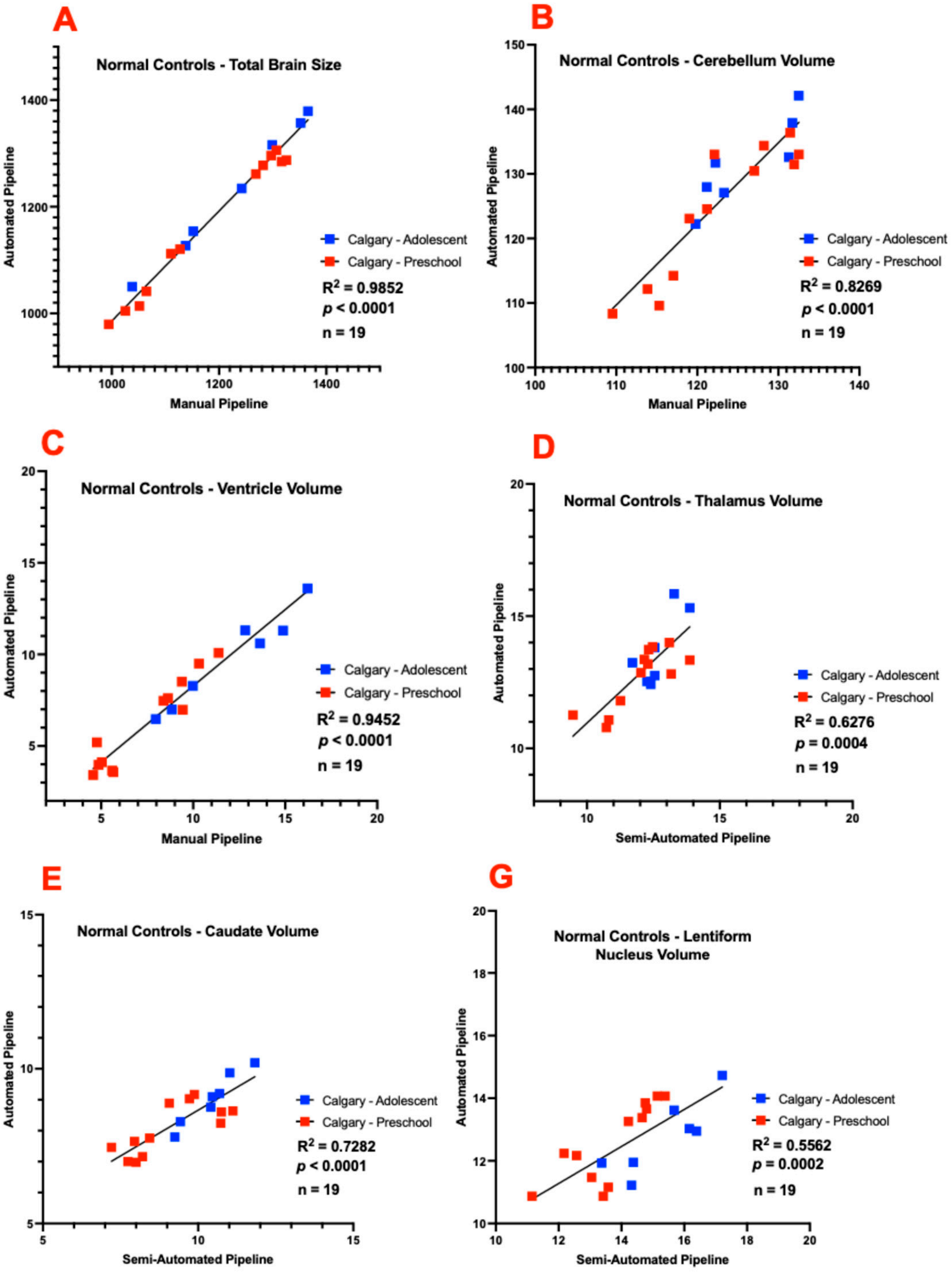

Figure S9.1. Correlations between volBrain's automated and the manual approach in neurotypical controls for the A.) Total brain volume B.) Cerebellum volume C.) Ventricle volume D.) Thalamic volume E.) Caduate volume and F.) Lentiform nucleus volume. Participants from the Calgary preschool MRI data set are shown in red and participants from the adolescent Calgary dataset are shown in blue. All volumes are shown in cm^3.

Juvenile GM1 Patients

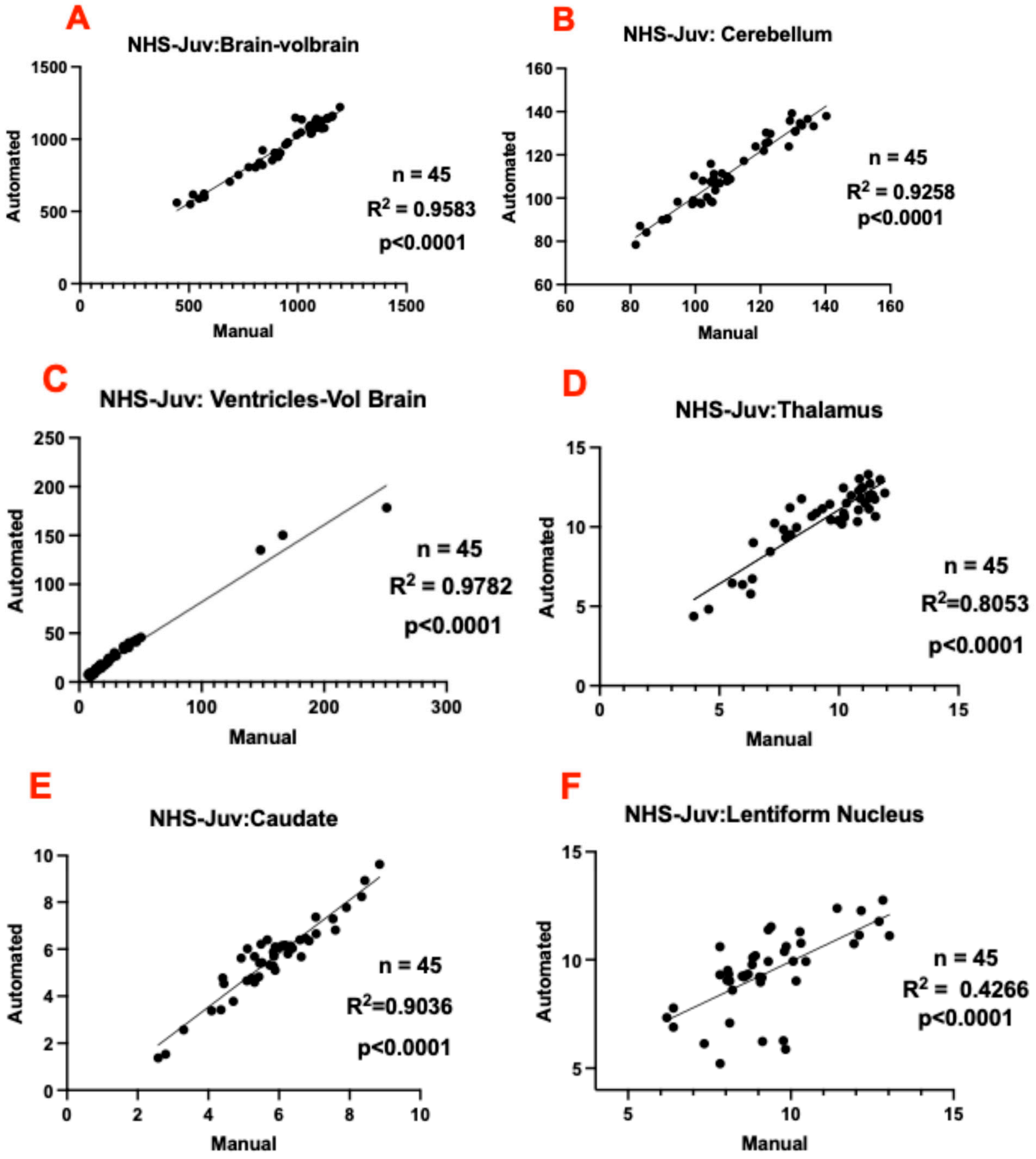

Figure S9.2. Correlations between volBrain's automated and the manual approach in natural history study (NHS) juvenile GM1 gangliosidosis patients for the A.) Total brain volume B.) Cerebellum volume C.) Ventricle volume D.) Thalamic volume E.) Caduate volume and F.) Lentiform nucleus volume. All volumes are shown in cm^3.

Late-Infantile GM1 Patients

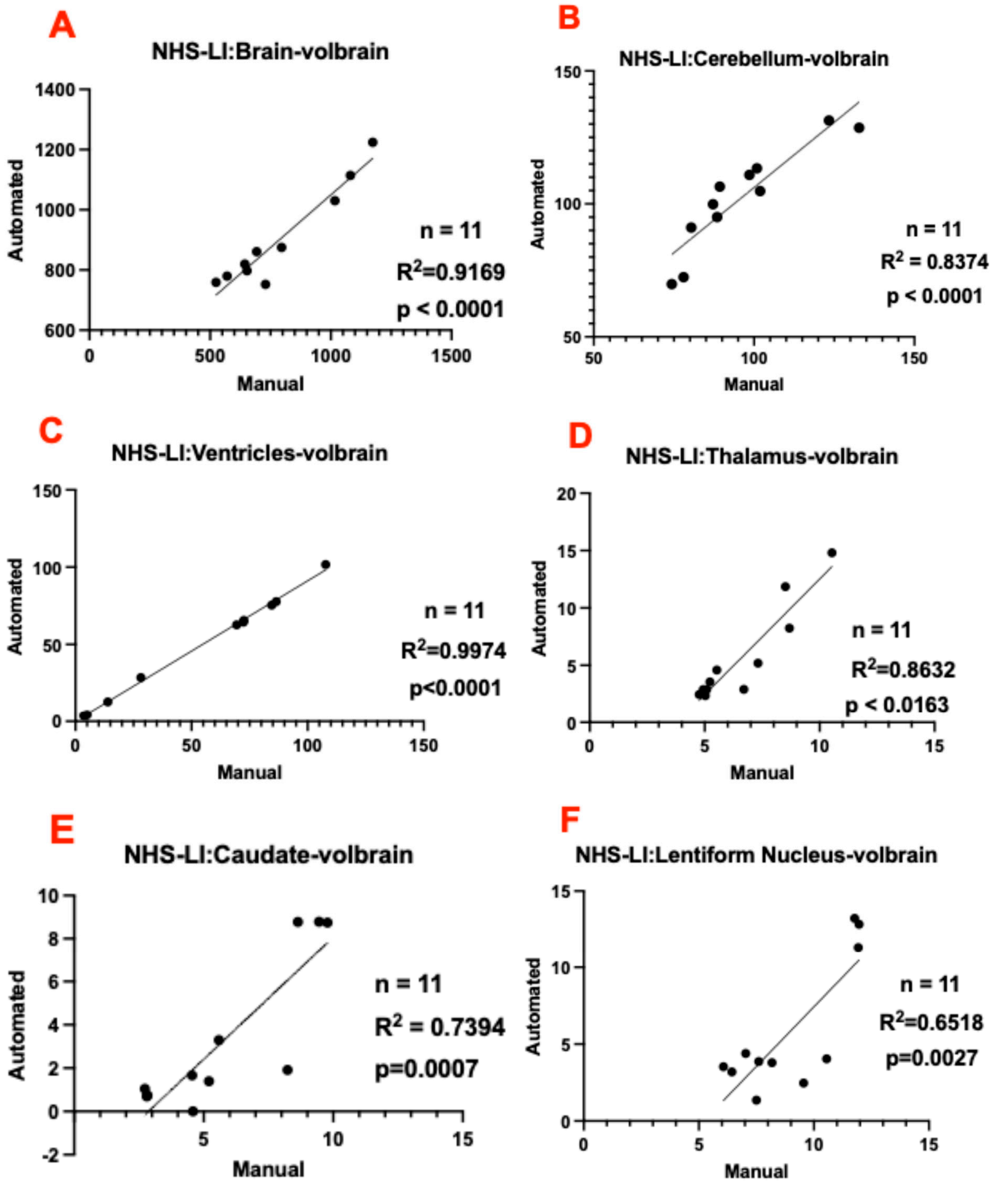

Figure S9.3. Correlations between volBrain's automated and the manual approach in natural history study (NHS) late-infantile GM1 gangliosidosis patients for the A.) Total brain volume B.) Cerebellum volume C.) Ventricle volume D.) Thalamic volume E.) Caduate volume and F.) Lentiform nucleus volume. All volumes are shown in cm^3.

S10: Correlations between Manual and FSL Segmentation

Neurotypical Controls

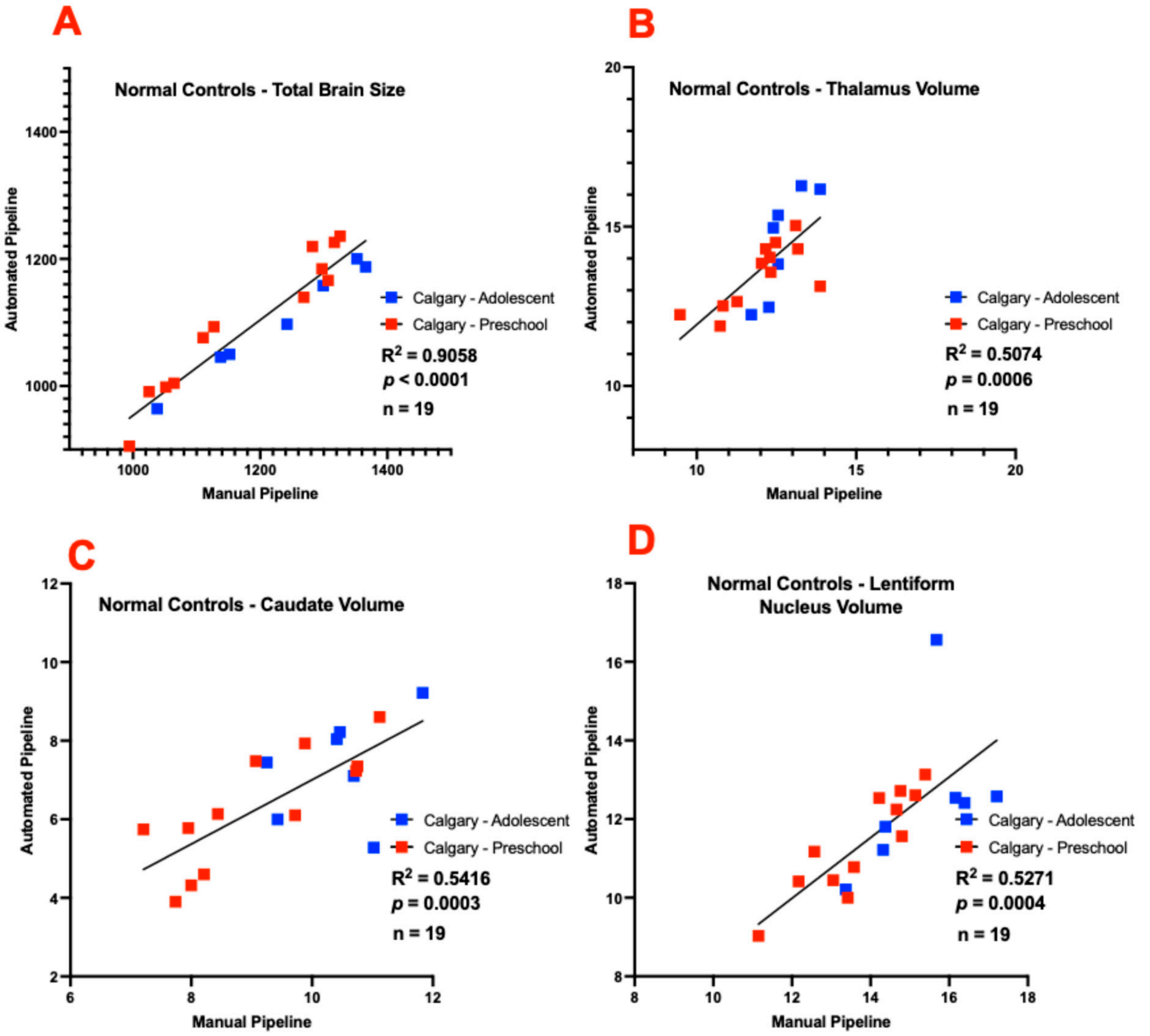

Figure S10.1. Correlations between FSL's automated and the manual approach in neurotypical controls for the A.) Total brain volume B.) Thalamic volume C.) Caduate volume and D.) Lentiform nucleus volume. Participants from the Calgary preschool MRI data set are shown in red and participants from the adolescent Calgary dataset are shown in blue. All volumes are shown in cm^3.

Juvenile GM1 Patients

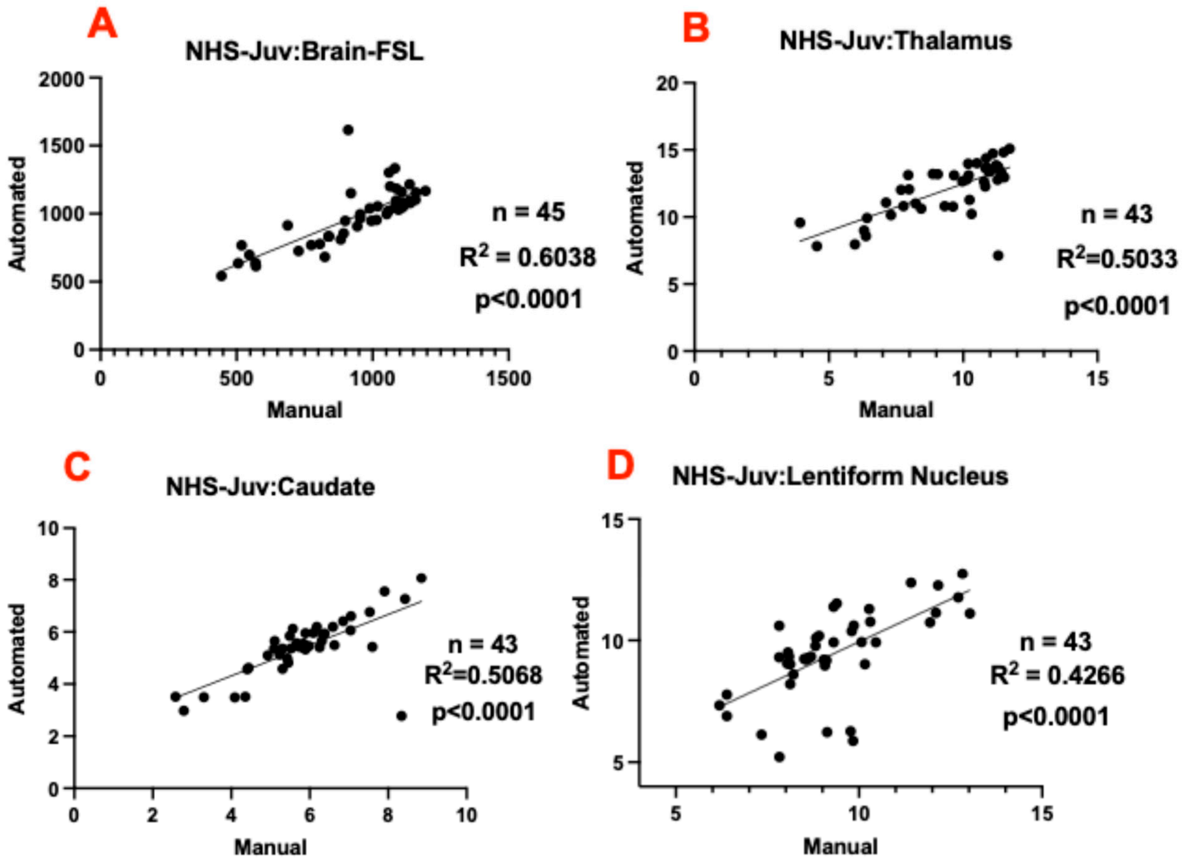

Figure S10.2. Correlations between FSL's automated and the manual approach in natural history study (NHS) juvenile GM1 gangliosidosis patients for the A.) Total brain volume B.) Thalamic volume C.) Caduate volume and D.) Lentiform nucleus volume. All volumes are shown in cm^3.

Late-Infantile GM1 Patients

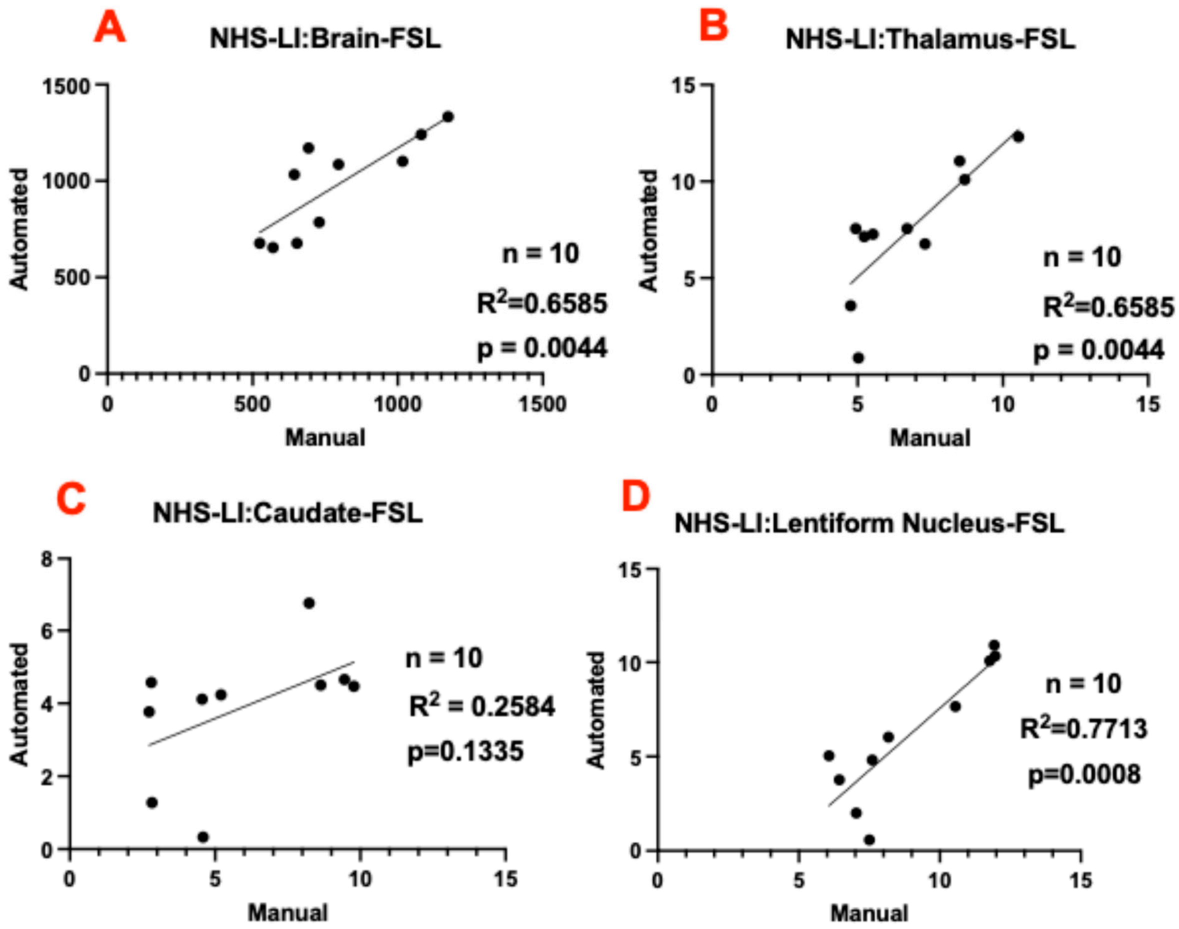

Figure S10.3. Correlations between FSL's automated and the manual approach in natural history study (NHS) juvenile GM1 gangliosidosis patients for the A.) Total brain volume B.) Thalamic volume C.) Caduate volume and D.) Lentiform nucleus volume. All volumes are shown in cm^3.

S11: Correlations between Manual and SPM Segmentation

Neurotypical Controls

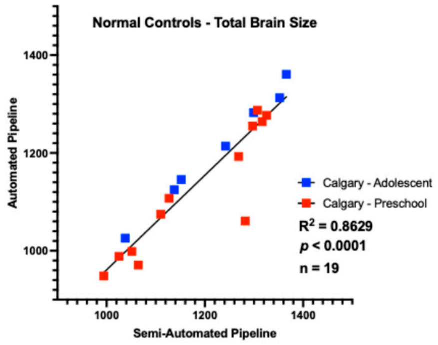

Figure S11.1. Correlations between SPM's automated and the manual approach in neurotypical controls for the total brain volume. Participants from the Calgary preschool MRI data set are shown in red and participants from the adolescent Calgary dataset are shown in blue. All volumes are shown in cm^3.

Juvenile GM1 Patients

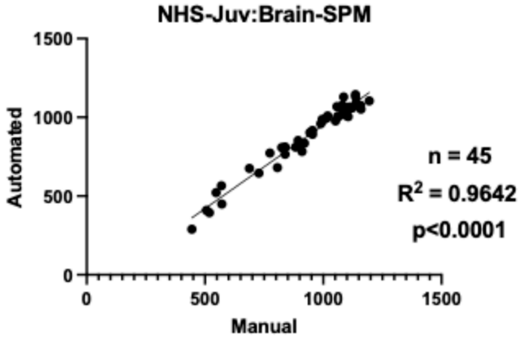

Figure S11.2. Correlations between SPM's automated and the manual approach in natural history study (NHS) juvenile GM1 gangliosidosis patients for total brain volume. All volumes are shown in cm^3.

Late-Infantile GM1 Patients

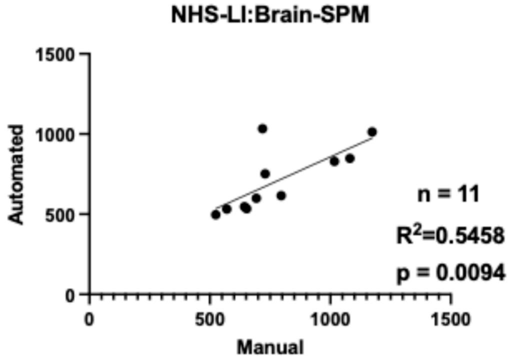

Figure S11.3. Correlations between SPM's automated and the manual approach in natural history study (NHS) late-infantile GM1 gangliosidosis patients for total brain volume. All volumes are shown in cm^3.

S12: Correlations between Manual and Headreco Segmentation

Neurotypical Controls

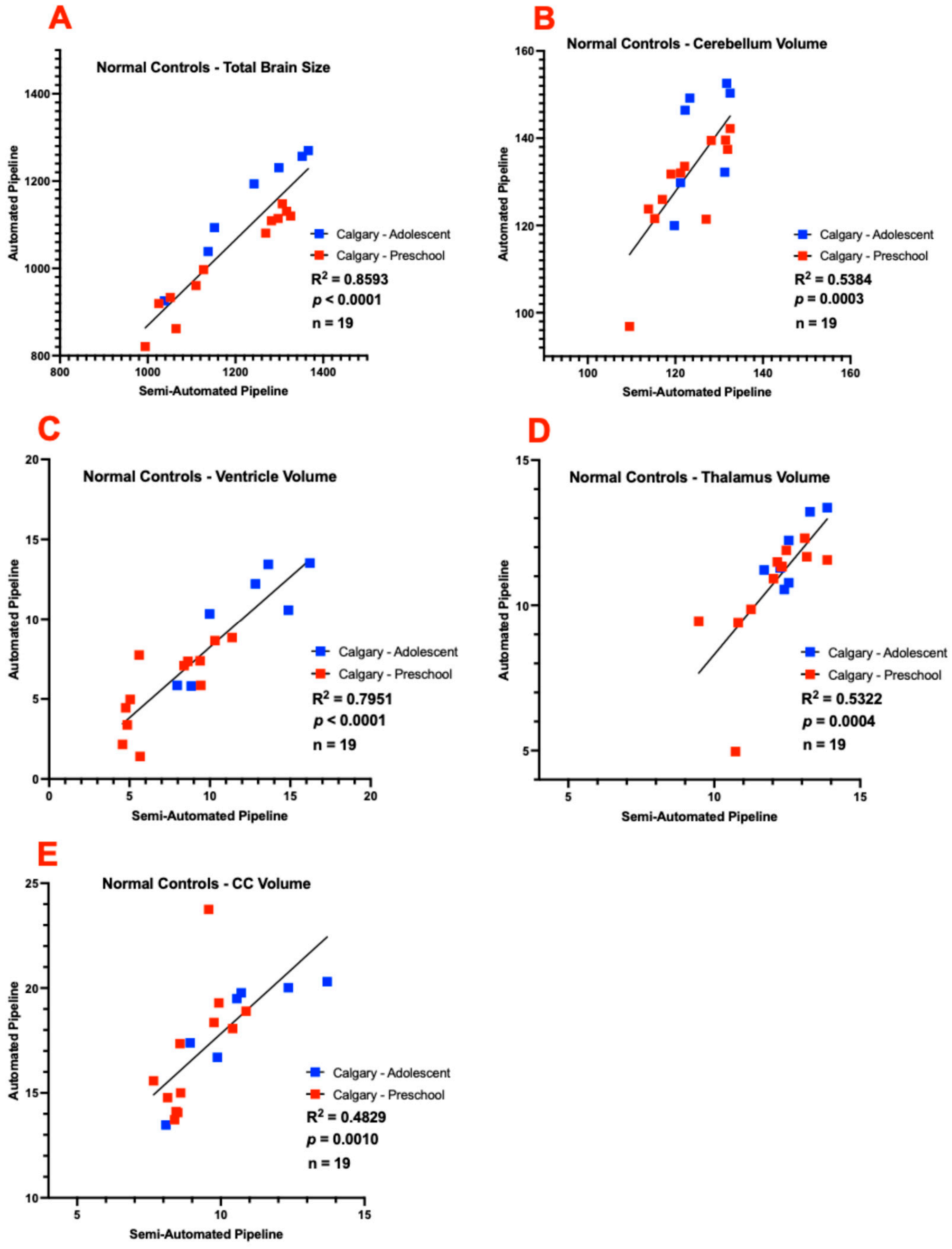

Figure S12.1. Correlations between Headreco's automated and the manual approach in neurotypical controls for the A.) Total brain volume B.) Cerebellum volume C.) Ventricle volume D.) Thalamic volume and E.) Corpus callosum volume. Participants from the Calgary preschool MRI data set are shown in red and participants from the adolescent Calgary dataset are shown in blue. All volumes are shown in cm^3.

Juvenile GM1 Patients

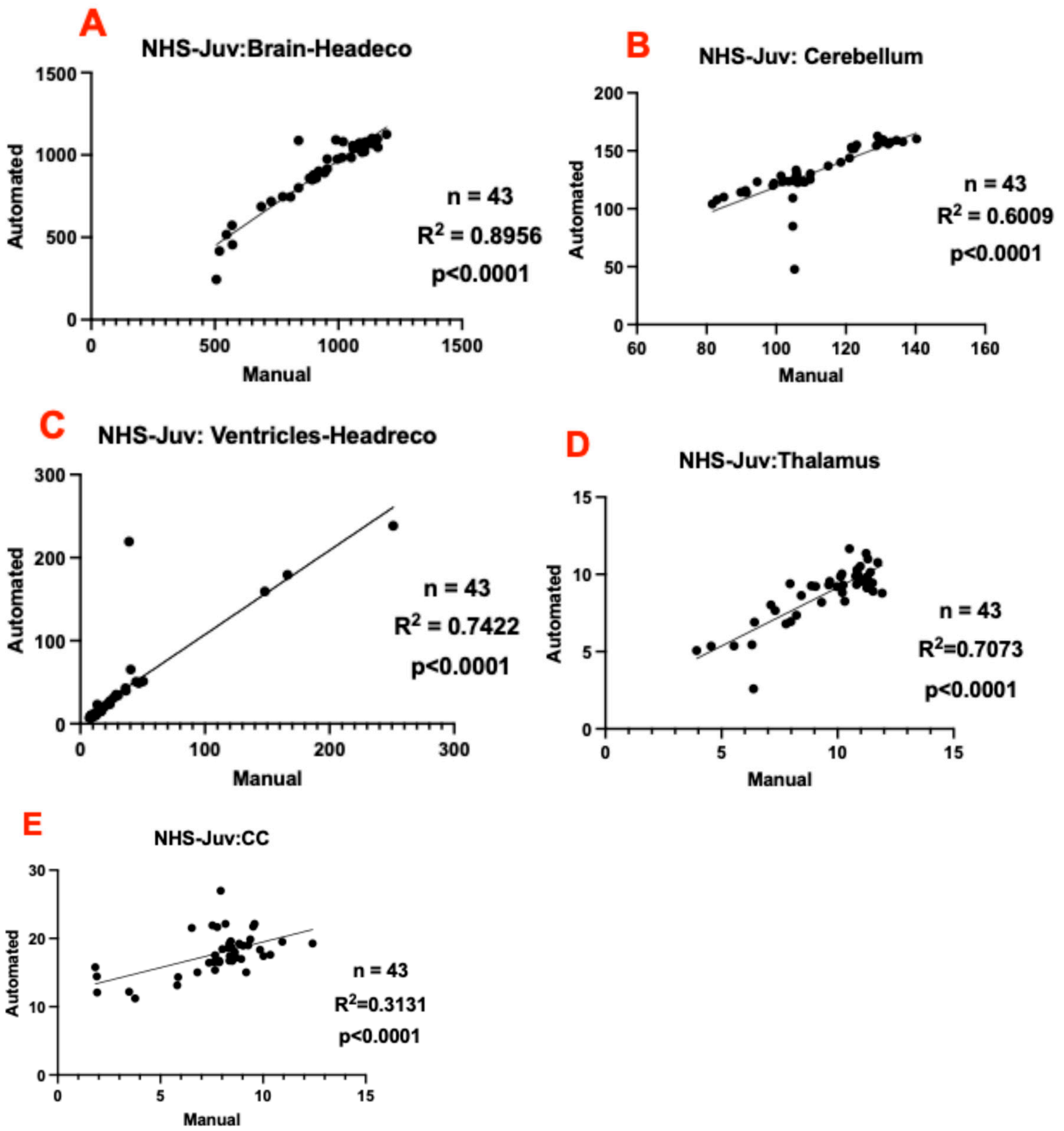

Figure S12.2. Correlations between Headreco's automated and the manual approach in natural history study (NHS) juvenile GM1 gangliosidosis patietns for the A.) Total brain volume B.) Cerebellum volume C.) Ventricle volume D.) Thalamic volume and E.) Corpus callosum volume. All volumes are shown in cm^3.

Late-Infantile GM1 Patients

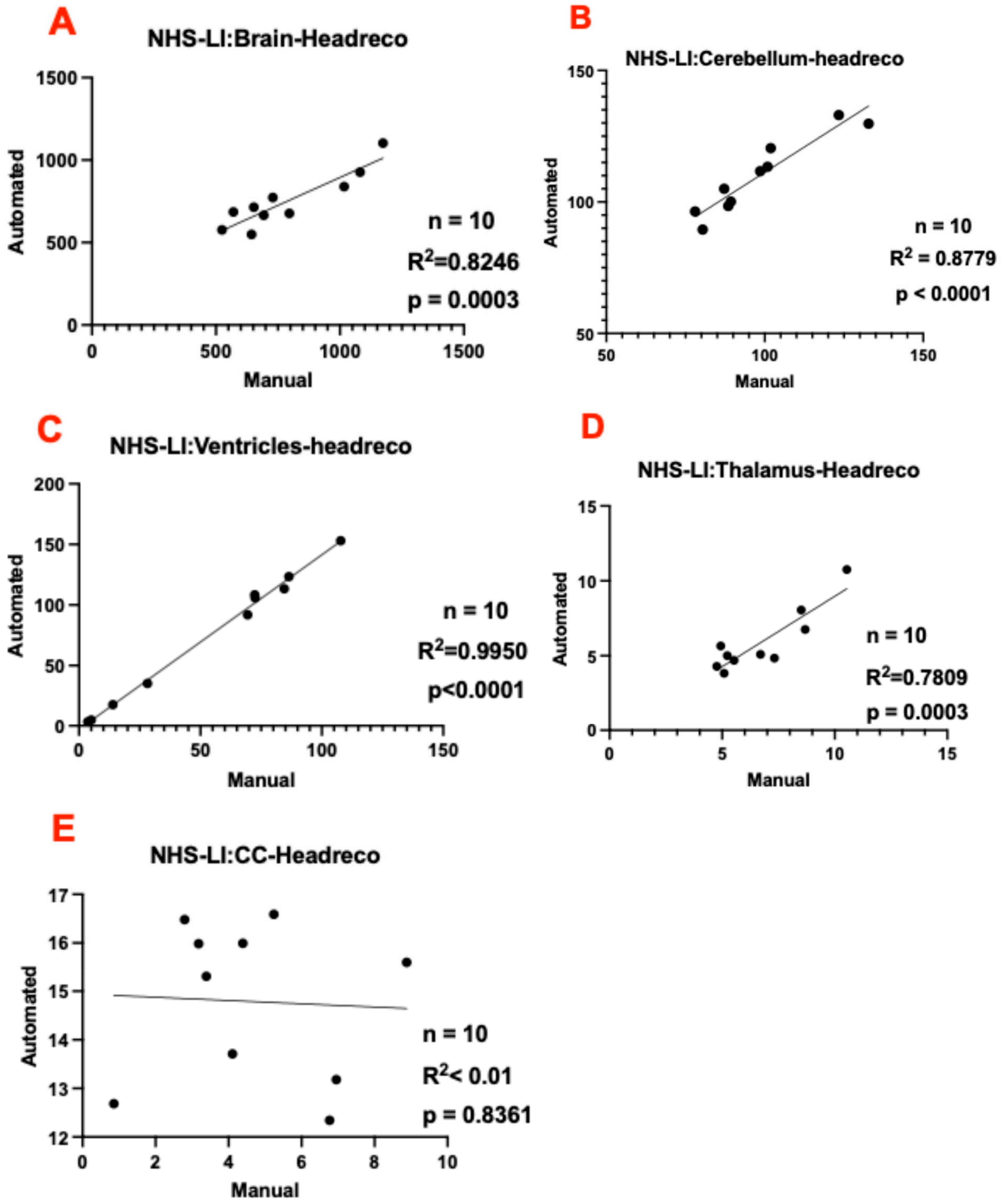

Figure S12.3. Correlations between Headreco's automated and the manual approach in natural history study (NHS) late-infantile GM1 gangliosidosis patietns for the A.) Total brain volume B.) Cerebellum volume C.) Ventricle volume D.) Thalamic volume and E.) Corpus callosum volume. All volumes are shown in cm^3.

S13: Slopes and Intercepts between Manual and Automated Segmentation

Table S13.1. Comparison of slopes and intercepts of the linear regression modeling between the 5 fully automated segmentation pipelines and the manual pipeline in the 7 different brain regions for the neurotypical controls. N/A are designations where the region was not calculated using the specified segmentation algorithm. The slope of the linear regression line is given in the top row, and the intercept is given in the bottom row for each cell. The 95% confidence interval for the slope and intercept shown in the brackets. For all linear regression modeling the automated volumetric calculations were along the y-axis, and the manual volumetric calculations were along the x-axis.

Structure	Freesurfer	FSL	volBrain	SPM	SimNIBS (SPM+CAT)
**Whole Brain**	0.99 [0.76, 1.23]<br>-99.32 [-380.9, 182.3]	0.75 [0.63, 0.88]<br>202.1 [52.83, 351.5]	1.03 [0.97, 1.10]<br>-44.99 [-122.8, 32.82]	0.97 [0.77, 1.16]<br>-6.21 [-243.7, 231.2]	0.98 [0.78, 1.19]<br>112.6 [-357.4, 132.2]
**Ventricles**	0.68 [0.40, 0.95]<br>2.77 [0.13, 5.41]	N/A	0.83 [0.73, 0.94]<br>-0.05 [-1.04, 0.95]	N/A	0.88 [0.65, 1.11]<br>-0.58 [-2.81, 1.64]
**Cerebellum**	1.27 [0.84, 1.71]<br>25.61 [-80.32, 27.75]	N/A	1.26 [0.96, 1.55]<br>-28.53 [-65.00, 7.93]	N/A	1.38 [0.73, 2.04]<br>-38.04 [-119.2, 43.11]
**Thalamus**	1.15 [0.60, 1.70]<br>0.34 [-6.39, 7.08]	0.87 [0.43, 1.30]<br>3.26 [-2.11, 8.62]	0.94 [0.57, 1.31]<br>1.55 [-3.00, 6.10]	N/A	1.21 [0.63,1.78]<br>-3.75 [-10.85, 3.35]
**Caudate**	0.89 [0.67, 1.10]<br>-0.39 [-2.47, 1.68]	0.82 [0.43, 1.20]<br>-1.18 [-4.90, 2.55]	0.59 [0.41, 0.78]<br>2.75 [0.96, 4.53]	N/A	N/A
**Lentiform Nucleus**	0.80 [0.54, 1.05]<br>2.55 [-1.06, 6.16]	0.77 [0.40, 1.15]<br>0.72 [-4.67, 6.12]	0.59 [0.32, 0.86]<br>2.13 [0.28, 8.08]	N/A	N/A
**Corpus Callosum**	0.09 [-0.08, 0.27]<br>2.04 [0.33, 3.74]	N/A	N/A	N/A	1.25 [0.59, 1.90]<br>5.38 [-1.05, 11.81]

Table S13.2. Comparison of slopes and intercepts of the linear regression modeling between the 5 fully automated segmentation pipelines and the manual pipeline in the 7 different brain regions for the juvenile GM1 patients. N/A are designations where the region was not calculated using the specified segmentation algorithm. The slope of the linear regression line is given in the top row, and the intercept is given in the bottom row for each cell. The 95% confidence interval for the slope and intercept shown in the brackets. For all linear regression modeling the automated volumetric calculations were along the y-axis, and the manual volumetric calculations were along the x-axis.

Structure	Freesurfer	FSL	volBrain	SPM	SimNIBS (SPM+CAT)
**Whole Brain**	0.85 [0.77, 0.95]<br>74.26 [-10.34, 158.9]	0.82 [0.63, 1.02]<br>215.7 [20.80, 410.5]	0.92 [0.86, 0.98]<br>92.83 [36.75, 148.9]	1.06 [1.00, 1.12]<br>-109.9 [-170.1, -46.67]	1.04 [0.92, 1.15]<br>-68.93 [-177.2, 39.38]
**Ventricles**	0.97 [0.92, 1.02]<br>0.80 [-0.40, 2.00]	N/A	0.79 [0.75, 0.83]<br>2.89 [0.88, 4.91]	N/A	0.91 [0.87, 0.95]<br>0.22 [-2.24, 2.68]
**Cerebellum**	1.00 [0.90, 1.11]<br>5.48 [-6.27, 17.24]	N/A	1.04 [0.95, 1.12]<br>-2.67 [-12.42, 7.09]	N/A	1.15 [0.86, 1.45]<br>3.55 [-29.67, 36.78]
**Thalamus**	0.63 [0.39, 0.88]<br>6.53 [4.09, 8.98]	0.71 [0.48, 0.93]<br>5.42 [3.30, 7.55]	0.94 [0.79, 1.08]<br>1.74 [0.38, 3.11]	N/A	0.76 [0.60, 0.91]<br>1.58 [0.09, 3.07]
**Caudate**	0.83 [0.70, 0.96]<br>0.54 [-0.29, 1.37]	0.59 [0.41, 0.77]<br>1.96 [0.86, 3.06]	1.14 [1.02, 1.26]<br>-1.02 [-1.72, -0.33]	N/A	N/A
**Lentiform Nucleus**	0.97 [0.69, 1.25]<br>0.39 [-2.28, 3.06]	0.70 [0.44, 0.96]<br>2.94 [0.51, 5.36]	0.70 [0.44, 0.96]<br>2.99 [0.51, 5.46]	N/A	N/A
**Corpus Callosum**	0.23 [0.14, 0.32]<br>0.79 [0.04, 1.54]	N/A	N/A	N/A	0.75 [0.40, 1.10]<br>11.96 [9.11, 14.81]

Table S13.3. Comparison of slopes and intercepts of the linear regression modeling between the 5 fully automated segmentation pipelines and the manual pipeline in the 7 different brain regions for the late-infantile GM1 patients. N/A are designations where the region was not calculated using the specified segmentation algorithm. The slope of the linear regression line is given in the top row, and the intercept is given in the bottom row for each cell. The 95% confidence interval for the slope and intercept shown in the brackets. For all linear regression modeling the automated volumetric calculations were along the y-axis, and the manual volumetric calculations were along the x-axis.

Structure	Freesurfer	FSL	volBrain	SPM	SimNIBS (SPM+CAT)
Whole Brain	0.32 [-0.04, 00.69]<br>657.2 [361.0, 953.4]	0.68 [-1.21, 2.57]<br>593.0 [-934.2, 2120]	0.70 [0.53, 0.87]<br>349.7 [209.4, 490.0]	0.68 [0.21, 1.15]<br>178.6 [-198.5, 555.8]	0.68 [0.42, 0.93]<br>217.8 [10.00, 425.6]
Ventricles	0.68 [0.35, 1.00]<br>7.70 [-13.27, 28.62]	N/A	0.91 [0.87, 0.95]<br>0.22 [-2.24, 2.68]	N/A	1.44 [1.36, 1.52]<br>-2.55 [-7.98, 2.87]
Cerebellum	0.63 [0.27, 0.99]<br>38.87 [3.25, 74.49]	N/A	0.98 [0.66, 1.31]<br>-8.23 [-23.71, 39.85]	N/A	0.85 [0.61, 1.09]<br>25.10 [1.70, 48.49]
Thalamus	0.97 [0.23, 1.71]<br>3.51 [-1.65, 8.66]	1.38 [0.57, 2.19]<br>-1.85 [-7.50, 3.80]	2.02 [1.41, 2.62]<br>-7.63 [-11.77, -3.50]	N/A	0.92 [0.55, 1.28]<br>-0.23 [-2.73, 2.28]
Caudate	0.70 [0.47, 0.94]<br>0.73 [-2.47, 2.27]	0.32 [-0.12, 0.77]<br>1.97 [-0.92, 4.86]	1.13 [0.62, 1.63]<br>-3.24 [-6.47, 0.00]	N/A	N/A
Lentiform Nucleus	1.22 [0.51, 1.94]<br>-3.26 [-9.94, 3.43]	1.32 [0.73, 1.90]<br>-5.59 [-10.95, -0.22]	1.55 [0.70, 2.41]<br>-8.08 [-15.96, -0.20]	N/A	N/A
Corpus Callosum	0.06 [-0.14, 0.26]<br>1.64 [0.60, 2.69]	N/A	N/A	N/A	-0.05 [-0.55, 0.45]<br>15.03 [12.56, 17.51]

S14: Supplementary References

1. National Human Genome Research Institute. Natural History of Glycosphingolipid Storage Disorders and Glycoprotein Disorders ClinicalTrials.gov identifier: NCT00029965. Updated August 7, 2024. Accessed August 12, 2024. https://clinicaltrials.gov/study/NCT00029965.
2. Reynolds JE, Long X, Paniukov D, Bagshawe M, Lebel C. Calgary Preschool magnetic resonance imaging (MRI) dataset. Data Brief. 2020 Jan 31;29:105224. doi: 10.1016/j.dib.2020.105224. PMID: 32071993; PMCID: PMC7016255.
3. Mah A, Geeraert B, Lebel C (2017) Detailing neuroanatomical development in late childhood and early adolescence using NODDI. PLoS ONE 12(8): e0182340. https://doi.org/10.1371/journal.pone.0182340.
